# Solvation-Tuned Photoacid as a Stable Light-Driven pH Switch for CO_2_ Capture and Release

**DOI:** 10.1021/acs.chemmater.3c02435

**Published:** 2023-12-20

**Authors:** Anna de Vries, Kateryna Goloviznina, Manuel Reiter, Mathieu Salanne, Maria R. Lukatskaya

**Affiliations:** † Electrochemical Energy Systems Laboratory, Department of Mechanical and Process Engineering, ETH Zurich, 8092 Zurich, Switzerland; ‡ Sorbonne Université, CNRS, Physico-Chimie des Électrolytes et Nanosystèmes Interfaciaux, PHENIX, F-75005 Paris, France; § Institut Universitaire de France (IUF), 75231 Paris, France

## Abstract

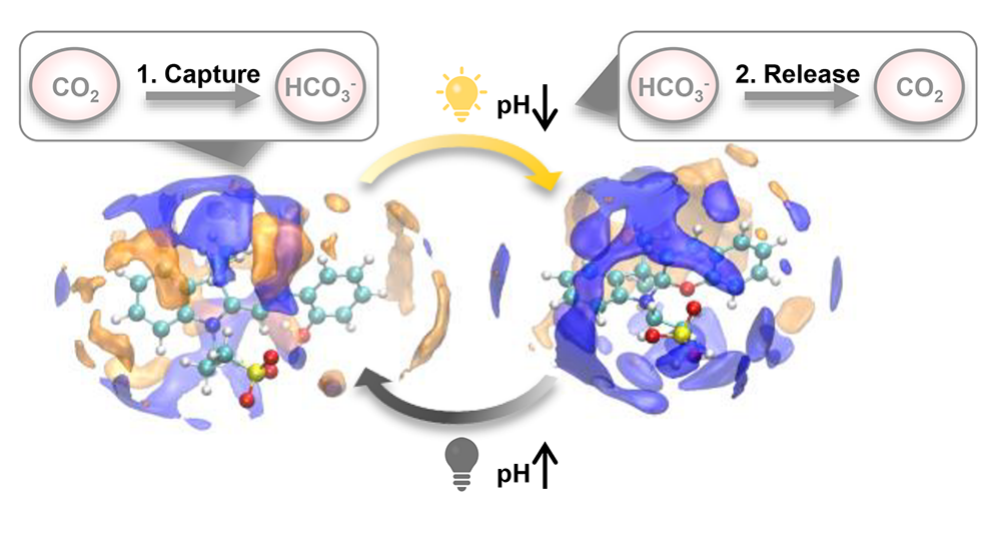

Photoacids are organic molecules that release protons under illumination, providing spatiotemporal control of the pH. Such light-driven pH switches offer the ability to cyclically alter the pH of the medium and are highly attractive for a wide variety of applications, including CO_2_ capture. Although photoacids such as protonated merocyanine can enable fully reversible pH cycling in water, they have a limited chemical stability against hydrolysis (<24 h). Moreover, these photoacids have low solubility, which limits the pH-switching ability in a buffered solution such as dissolved CO_2_. In this work, we introduce a simple pathway to dramatically increase stability and solubility of photoacids by tuning their solvation environment in binary solvent mixtures. We show that a preferential solvation of merocyanine by aprotic solvent molecules results in a 60% increase in pH modulation magnitude when compared to the behavior in pure water and can withstand stable cycling for >350 h. Our results suggest that a very high stability of merocyanine photoacids can be achieved in the right solvent mixtures, offering a way to bypass complex structural modifications of photoacid molecules and serving as the key milestone toward their application in a photodriven CO_2_ capture process.

## Introduction

**Scheme 1:**
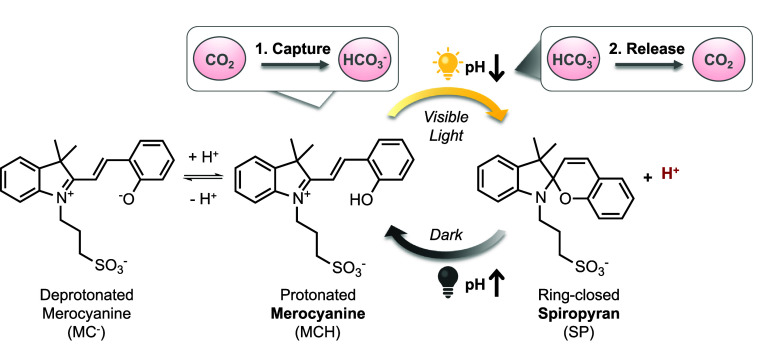
Simplified Photochemistry of Protonated Merocyanine Photoacid and Light-Driven CO_2_ Capture–Release Process

Proton transfer plays a fundamental role in regulating (electro)chemical processes relevant for catalysis,^1−3^ energy,^4^ CO_2_ storage,^5,6^ and many others.^7−10^ Therefore, noninvasive external control of the pH is highly desirable for the modulation of proton-coupled (electro)chemical reactions and equilibria. A unique class of molecules that are capable of generating H^+^ on demand upon illumination are called photoacids. Photoacids are organic molecules that possess higher acidity in their light-induced excited state compared to their ground state in darkness.^1^ The proton-transfer for these compounds is highly reversible: photoacids release a proton to their environment under excitation by UV or visible light, while in darkness, they return to the ground state by thermal relaxation and recombining with a proton. Metastable photoacids (mPAHs) are a subclass of photoacids that can alter the pH of a solution through proton release as a result of photoisomerization. Such a mechanism allows mPAHs to remain in their deprotonated state for 9–10 orders of magnitude longer than other types of photoacids.^11^ The relaxation at the order of seconds–minutes makes it possible for released protons to diffuse away from their counterions and can thus reversibly alter the bulk pH in a solution.^1,12^ For example, protonated merocyanine (MCH) photoacids, the archetype of mPAHs, undergo *trans*-to-*cis* isomerization under UV or visible light illumination, leading to a proton release, followed by a ring-closing reaction to spiropyran (SP) (Scheme 1).^1,12,13^ As a result, bulk solution pH can drop by several units within seconds and return to the initial pH in darkness within minutes.^1,12,14,15^ As mPAHs provide remote pH-control of the medium, achievable by a moderate intensity of visible light, they have been demonstrated in numerous applications,^16,17^ among them are functionalized surfaces such as membranes^18^ and sensors,^19^ acid-catalyzed reactions such as drug delivery,^20,21^ volume-change of hydrogels,^12,22^ control of physicochemical properties of polymers such as conductivity with light,^23,24^ reversible nanoassembly of DNA,^25^ and molecular machines.^26,27^

Recently, there has been a growing interest in employing photoacids for CO_2_ capture applications.^28−34^ This approach harnesses a photodriven pH change, offering an attractive alternative to the energy-intensive pressure and temperature swings that are traditionally used for sorbent regeneration.^35^ In the process that employs photoacids, CO_2_ is first captured by an alkaline solution (sorbent) containing protonated photoacid, leading to the formation of bicarbonate (HCO_3_^–^).^36−38^ Next, to regenerate, the sorbent solution is exposed to light, triggering the release of protons followed by bicarbonate protonation, which in turn releases gaseous CO_2_.^28^ Finally, in the dark, the photoacid reverts to its protonated state, leading to an overall increase in pH and thereby regenerating the sorbent, completing the capture-release cycle (Scheme 1).

However, these practical applications of photoacids are challenged by insufficient stability of mPAH in aqueous media and limited solubility.^39−41^ In water, more than half of MCH undergoes hydrolysis along the bridging C=C bond within the first 24 h.^39^ Meanwhile, low solubility constraints the magnitude of the pH drop, especially for solutions that are buffered, such as in CO_2_ capture applications.^42^ Main efforts to improve the chemical stability and solubility were focused on modifying the chemical structure of merocyanine by adding electron donating and withdrawing substituents to the conjugated system.^14−16,43,44^ Other approaches include postsynthesis modifications such as confinement of photoacid molecules into coordination cages.^45−47^ While with these approaches the chemical half-life can be improved from 16 h up to several days,^39^ for real applications, such as CO_2_ capture, it is necessary to demonstrate a long-term cycling stability.^48^ However, to the best of our knowledge, demonstration of prolonged light cycling stability with such molecular approaches is still limited to a maximum of 17 h of operation.^25^ Stability of photoacids is known to be affected by the solvent environment,^1,13^ where higher stability can be achieved in nonaqueous solutions (e.g., methanol,^44,49−51^ DMF,^51^ DMSO^44,51−53^). In aprotic solvents, higher stability is enabled by natural suppression of the hydrolytic degradation pathway.^13^ However, the proton release of the photoacids is practically irreversible in aprotic solvents in contrast to protic ones.^1,52,54^ To enable reversible pH change with photoacids, solvents or solvent mixtures with an extended H-bonding network are necessary to facilitate proton transfer back to the photoacid in the darkness.^54^

In this work, we probe the stability and solubility of photoacids in water-DMSO mixtures. DMSO was selected as a cosolvent because it offers at least 2 orders of magnitude higher photoacid solubility and stability compared to water.^13^ Potentially, other aprotic cosolvents can offer the same benefits of high solubilization power and hydrolysis inhibition as DMSO.^55−57^ Further, in water–DMSO mixtures, freezing occurs at much lower temperatures (down to −140 °C).^58,59^ As the efficiency of MCH pH-switching scales inversely with temperature,^60^ incorporation of photoacids in water–DMSO mixtures would enable an improved magnitude and temperature range for such pH switches.^60^ Additionally, low toxicity^61^ and vapor pressure (0.051 kPa)^62^ of DMSO are particularly beneficial for long-term operation that is necessary in continuous processes such as CO_2_ capture.^63−65^

Herein, we show that merocyanine-type photoacids in water–DMSO solvent mixtures can achieve both high reversibility of the proton release and high chemical stability and solubility. We have selected a classical protonated merocyanine photoacid in water–DMSO mixtures as a model system as it is the most-studied structure among metastable photoacids.^12,39^ We demonstrate that solubility and pH modulation under illumination can be enhanced by 1 order of magnitude in solvent mixtures compared to pure water. Furthermore, we show that the stability of MCH can be dramatically extended in the solvent mixtures, prolonging pH cycling for over 350 h. By means of classical molecular dynamics (MD) simulations with polarizable force fields, we reveal the crucial role of the photoacid solvation environment in the observed enhancements. Finally, as a proof-of-concept, we show that a cosolvent approach can also be applied to benefit light-driven CO_2_ release.

## Results and Discussion

### Photoswitching Behavior of Merocyanine Photoacid in Water–DMSO Mixtures

**Figure 1:**
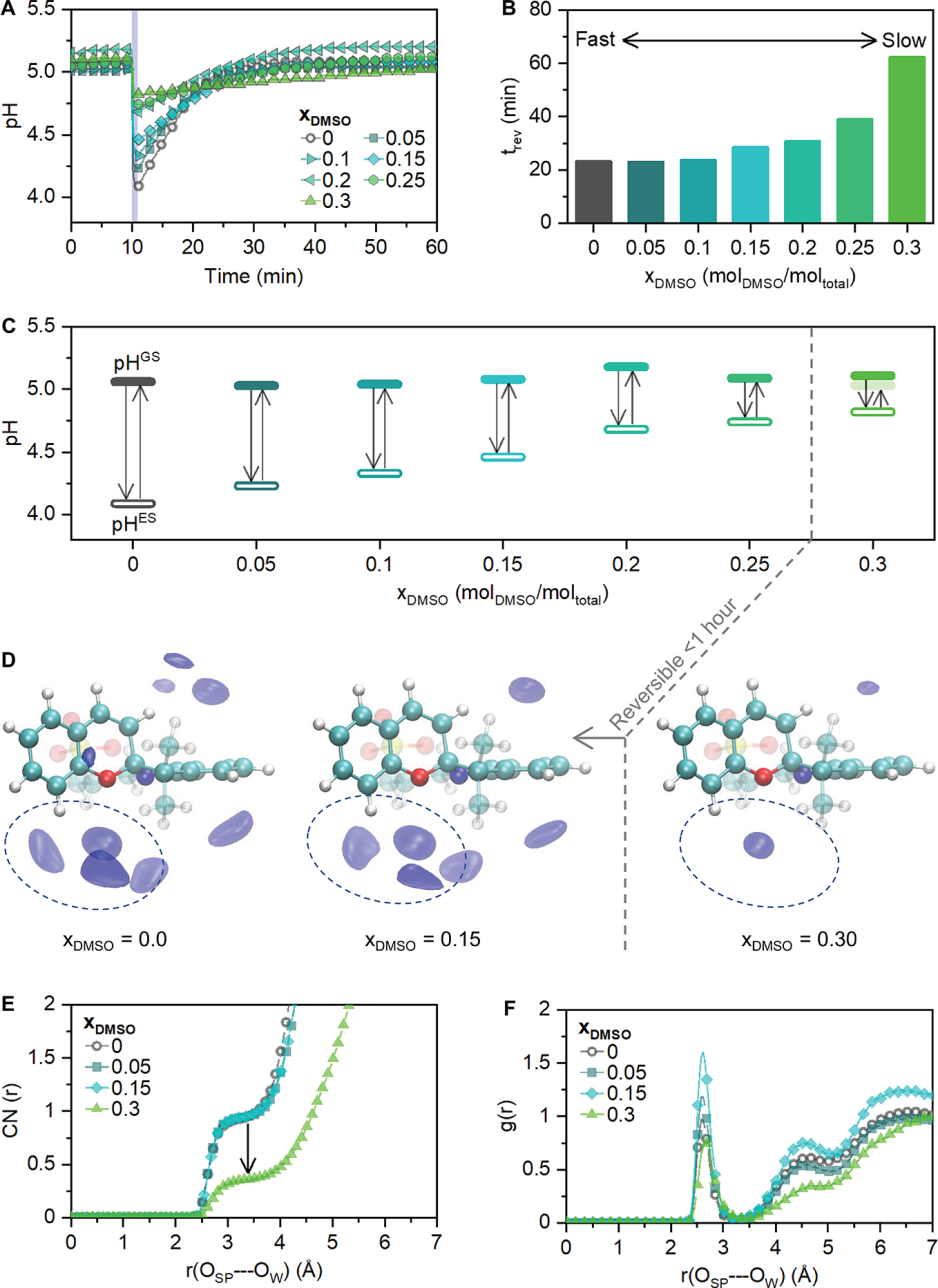
(A) pH-jump after illumination (at *t* = 10 min) of 0.08 mM mPA*H* at different DMSO mole fractions (*x*_DMSO_), (B) reversibility time upon relaxation to MCH in darkness, and (C) pH-jump as a function of *x*_DMSO_, where the dashed line indicates that at *x*_DMSO_ ≥ 0.3, the original pH cannot be recovered within 1 h of darkness. (D) Spatial distribution functions of oxygen atoms of water (blue isosurface) around the SP molecule, cut at the absolute density of 95 nm^–3^. (E) Coordination numbers of oxygen atoms of water around the oxygen atom of the SP molecule and (F) radial distribution functions of oxygen atoms of water around the oxygen atom of the SP molecule.

To analyze the role of DMSO on photoacid properties, we first characterized the pH-jump, ΔpH, and reversibility time, *t*_rev_ (Figure 1). The pH-jump (eq 1) is the difference between the solution pH when the photoacid is in the ground state in darkness (pH^GS^) and in the metastable state during illumination (pH^MS^).^13^ The pH values in different solvent mixtures should be treated as trends rather than absolute values, due to the change in activity coefficients and liquid junction potentials in the presence of nonaqueous solvents.^66−68^ If absolute pH values across different solvent compositions are required, they can be obtained following the procedures by Radtke et al. and Heering et al.^69,70^ The reversibility time (*t*_rev_) is the time it takes for the solution pH to return to the initial pH^GS^ value upon thermal relaxation.
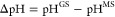
1

For these measurements, solutions with equal photoacid concentrations (0.08 mM) were prepared (preparation details described in Methods). As shown in Figure 1A–C, in pure water, a pH-jump of 0.8 units is observed. However, an increase in the DMSO mole fraction (*x*_DMSO_) leads to a decrease in the magnitude and efficiency of the pH-jump (Figure 1A–C). At *x*_DMSO_ = 0.3, the pH-jump is limited to 0.3 units due to less protons being released. Similarly, the time for the pH to reverse to the original pH^GS^ (*t*_rev_) gradually increases as a function of *x*_DMSO_ (Figure 1 A,B) and at *x*_DMSO_ ≥ 0.3, the pH-jump is no longer fully reversible within 1 h (since for applications the fast pH modulation is desired,^1,16,39,71^ only a *t*_rev_ of up to 1 h was considered). It is important to note that the proton release and reversibility time can be significantly affected by the setup (e.g., light power, wavelength, path-length, stirring efficiency), and results therefore may differ between reports.^13^

We used MD simulations to understand the impact of different solvent ratios in water–DMSO mixtures on the reversibility of the proton transfer to spiropyran (the ring-closed form that is reached in the metastable state, Scheme 1). The structure can be analyzed through the calculation of radial distribution functions, which are defined as the probability of finding atoms at a particular distance around a central reference atom and from which coordination numbers can be derived. Our results, shown in Figure 1E,F, indicate that the solvation environment of spiropyran changes notably as the DMSO content increases. In particular, the fraction of water molecules coordinating with the oxygen atom of spiropyran drops significantly at higher DMSO concentrations, from 0.94 (*x*_DMSO_ ≤ 0.15) to 0.35 at higher DMSO content (*x*_DMSO_ = 0.3). This behavior is also reflected in the radial distribution functions: the intensity of the first peak initially increases with lowering water content (dilution effect) but dramatically decreases once *x*_DMSO_ = 0.3 is achieved (Figure 1F). Similarly, at *x*_DMSO_ = 0.3 we observe a diminishment of the second solvation layer of water (formed at ∼5.1 Å) visible from spatial distribution functions (Figure 1D), leading to the coordination number decrease by a factor of 3 (from 5.0 at *x*_DMSO_ = 0.15 to 1.5 at *x*_DMSO_ = 0.3), which shows the absence of a water nanocluster in the vicinity of the oxygen atom of spiropyran. These changes in the solvation environment limit the possibility of passing protons through the hydrogen bonding network of water to spiropyran, blocking the ring-opening reaction (SP → MCH, Scheme 1).

### Enhanced Chemical Stability by Solvation Environment Tuning

**Figure 2:**
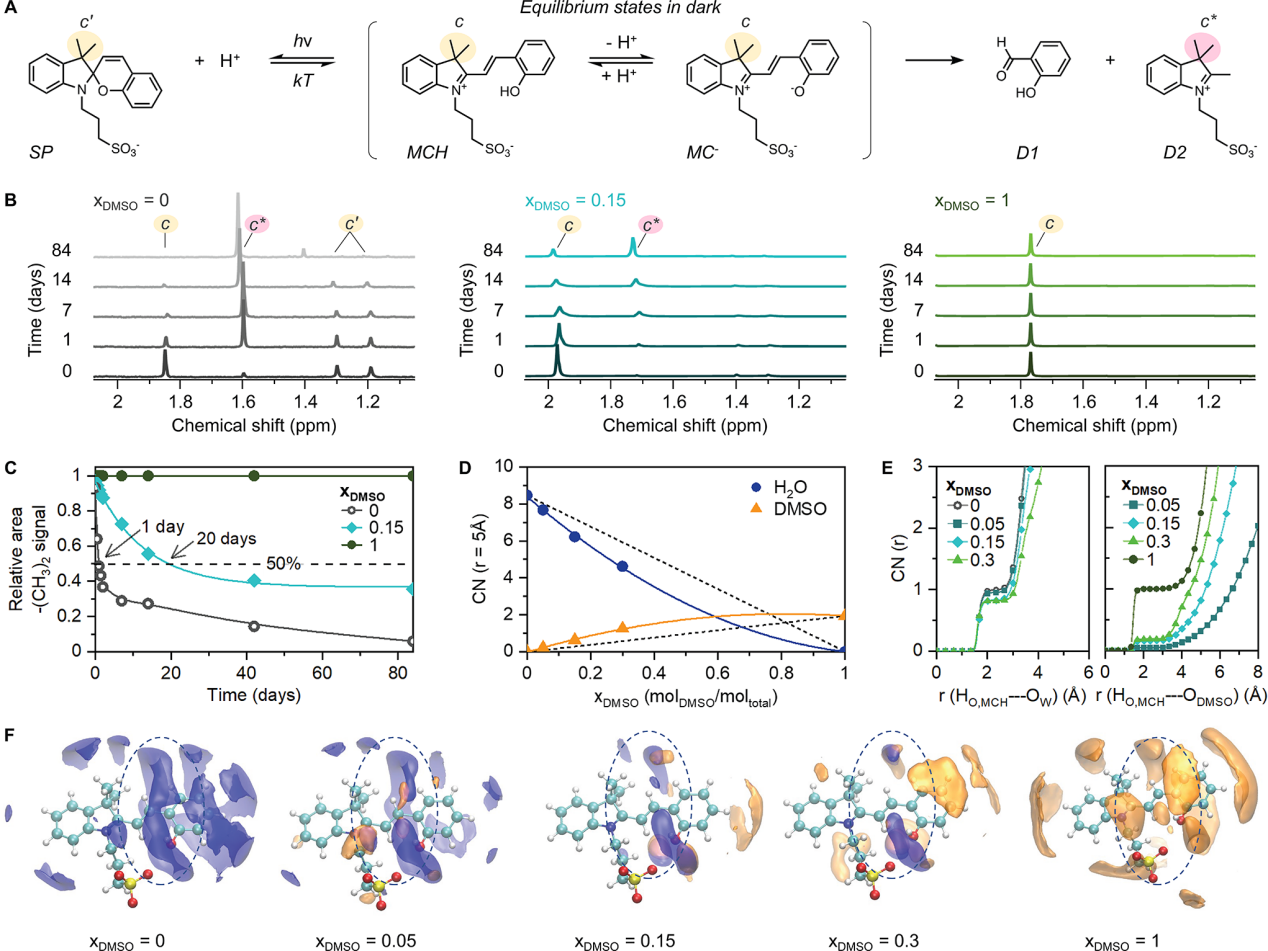
(A) Schematic of the equilibrium between ring closed spiropyran (SP), protonated and deprotonated merocyanine (MCH and MC^–^ respectively), and degradation products salicylaldehyde (D1) and indolium (D2). (B) ^1^H NMR spectra of mPA*H* in D_2_O (0.2 mM), *x*_DMSO-*d*_ = 0.15 (5.3 mM) and DMSO-*d*_6_ (17.5 mM) from left to right. (C) Chemical degradation over 12 weeks was obtained from ^1^H NMR signals. (D) Coordination numbers of oxygen atoms of water (blue) and DMSO (orange) around the double bond of the MCH molecule, evaluated at a distance of 5 Å, as a function of DMSO mole fraction. (E) Coordination numbers of oxygen atoms of water (left) and DMSO (right) around the hydrogen atom of the hydroxyl group of the MCH molecule. (F) Spatial distribution functions of oxygen atoms of water (blue isosurface) and DMSO (orange isosurface) around the MCH molecule. Isosurfaces are cut at the absolute density of 60 nm^–3^ for water and 4.5, 7.5, 13, and 14 nm^–3^ for *x*_DMSO_ = 0.05, 0.15, 0.30, and 1, respectively.

Next, we studied the chemical stability of MCH in water–DMSO mixtures. The degradation happens via a nucleophilic attack of water to the bridging C=C bond, leading to formation of salicylaldehyde (D1) and 2,3,3-trimethyl-1-(3-sulfonatepropyl)-3*H*-indolium (D2), as shown in Figure 2A.^39^ For *x*_DMSO_ = 0, 0.15, and 1, using ^1^H NMR spectroscopy, we tracked evolution of the peaks corresponding to MCH and products of its hydrolysis over a course of 12 weeks (Figure 2B). In water, the photoacid hydrolyzes rapidly: peaks from D2 (singlet peak at ∼1.6 ppm denoted as c*, (CH_3_)_2_-groups) are already present in the as-prepared sample (0 days), and the intensity increases sharply within the first 24 h. Concurrently, a steady decrease in intensity is observed for peaks corresponding to (CH_3_)_2_ groups of equilibrium forms of the unhydrolyzed photoacid: MCH/MC^–^ (∼1.85 ppm, singlet, c) and SP (∼1.2 and ∼1.3 ppm, doublet, c′). Analysis of the hydrolysis rates in water reveals that half of the mPAH is degraded within 1 day (Figure 2C). In contrast, in pure DMSO, no degradation products were detected even after 12 weeks: integrals for (CH_3_)_2_-groups of MCH/MC^–^ at ∼1.77 ppm remain stable over time, while the SP form is not observed (absence of the doublet at <1.4 ppm in Figure 2B). Meanwhile, in the mixture of both solvents (*x*_DMSO_ = 0.15), the hydrolysis is significantly suppressed (Figure 2C) with half of the photoacid concentration still present after 20 days.

Using MD simulations, this enhanced stability of merocyanine in water–DMSO mixtures compared to pure water can be explained from a structural point of view: in the first coordination shell of the bridging double bond of MCH, water molecules are progressively replaced by DMSO nanodomains with an increase in the DMSO content, as shown in Figure 2F. In particular, we observe higher coordination numbers for DMSO and lower ones for water than expected from ideal mixing (dashed line in Figure 2D).

This excess of DMSO points to preferable solvation of the double bond by the organic solvent. As a result, at *x*_DMSO_ = 0.3, the coordination number of the oxygen atom of DMSO reaches 65% of the maximum value in pure DMSO, preventing water species from approaching and reacting with the double bond. In the hydrolysis mechanism, the phenol/phenolate fragment of MCH plays an important role: Andreasson et al.^41^ showed that the phenolate oxygen (O^–^) of MC^–^ coordinates the water molecule approaching the bridging double bond, favoring water dissociation, and the O^–^ then stabilizes the formed proton.

The addition of water to the nondissociated OH group of MCH requires overcoming a significantly higher energy barrier, making this reaction path less probable. Formation of the metastable MC^–^ anion from the stable MCH form requires the proximity of water molecules to the phenol moiety. The presence of DMSO can perturb the H-bonding network, hampering the proton transfer from the solute to the solvent. MD simulations show that with the increase of the DMSO content, the coordination number of oxygen atoms of water around the hydroxyl group of MCH (measured at ∼2.35 Å from the H atom) decreases from 1.0 (*x*_DMSO_ = 0) to 0.82 (*x*_DMSO_ = 0.15) and remains almost unchanged up to *x*_DMSO_ = 0.3. Water molecules are replaced by those of DMSO since the inverse trend in coordination numbers is observed for the oxygen atoms of DMSO (Figure 2E). Even though only 0.18 O_DMSO_ (*x*_DMSO_ = 0.15) can be found around the OH group, it seems to already be sufficient to hinder the dissociation of the MCH, consequently stabilizing MCH in the solution.

### Optimizing Solubility and Stability for Long-Term Operation

**Figure 3:**
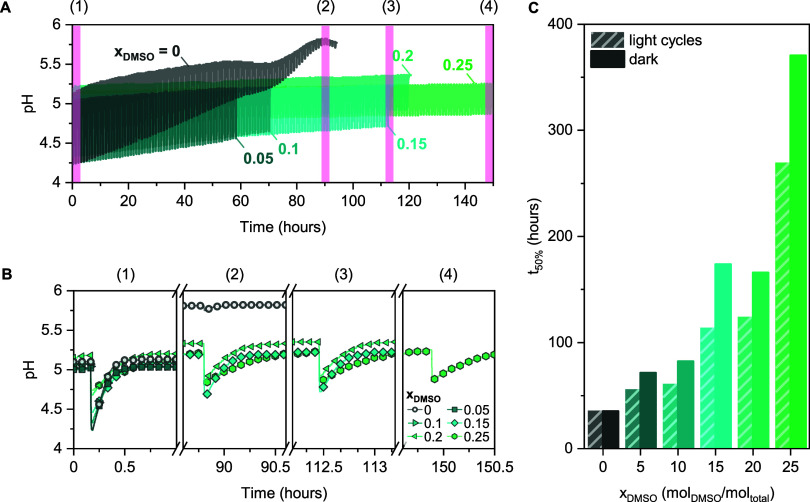
(A) Photochemical stability upon light cycles of 0.08 mM mPA*H* at different *x*_DMSO_ values where data is shown until 50% proton release degradation, with the exception of *x*_DMSO_ = 0 and 0.25 for which data is shown until 99 and 32% degradation, respectively. (B) pH-jump corresponding to selected regions in (A) at 0 (1), 90 (2), 113 (3), and 150 (4) h. (C) Time at which half of the protons are released during pH-jump compared to the first cycle (*t*_50%_).

Degradation of the mPAH can be further accelerated with illumination: repeated illumination or photoisomerization can lead to photobleaching.^72^ So far, photochemical stability has only been reported in water for MC-type photoacids up to 17 h of total operation.^25,39^ To probe the photochemical stability, we measured the pH of the photoacid solutions under light modulation (1 min light, 58 min dark, full procedure can be found in Methods). As can be seen in Figure 3A,B, the photochemical stability follows the same trend as chemical stability, as it increases with DMSO fraction: solutions at *x*_DMSO_ = 0.25 lost only 32% of the pH-jump magnitude after 150 h of cycling in contrast to the case of pure water, where 53% was already lost after 24 h. Faster degradation was observed for the solutions that were subject to light cycling compared to those kept in darkness (Figure 3C and Supporting Information S3).

**Figure 4:**
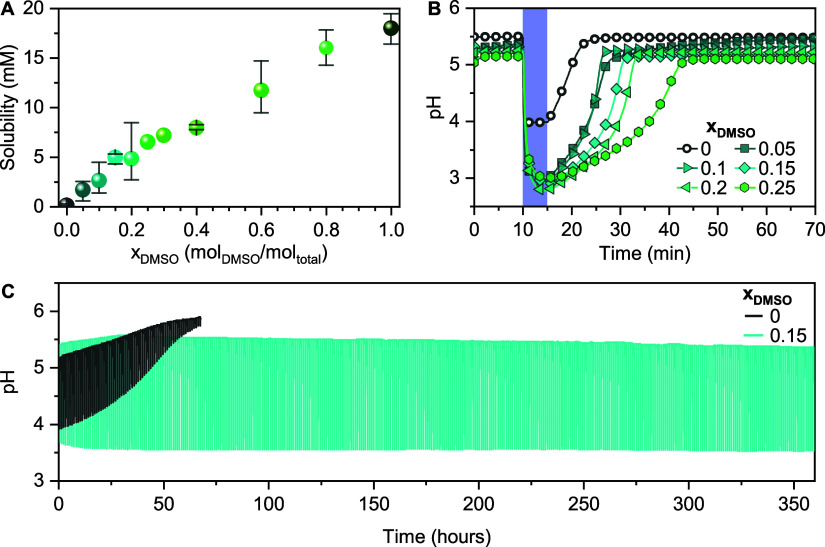
(A) Solubility curve of merocyanine photoacid in water–DMSO mixtures and (B) pH change after illumination of saturated mPAH (at *t* = 10 min) at different *x*_DMSO_ values. (C) Long-term stability of 0.2 mM mPA*H* in water and 5.3 mM in *x*_DMSO_ = 0.15, cycling by 1 min light and 58 min darkness.

Despite a notable improvement in chemical and photochemical stability of photoacid in water-DMSO mixtures (Figures 2 and 3), for the same concentration of mPAH, smaller pH-jumps were observed with higher *x*_DMSO_ (Figure 1A,C). In water, an increase in mPAH concentration leads to higher pH-jumps because more photoacid is present to release protons (Figure S10). Meanwhile, we show that in water–DMSO mixtures, an order of magnitude enhanced solubility can be achieved (Figure 4A, Table S2). Our computational results (Figure 1D) show that H_2_O primarily solvates polar regions and DMSO solvates nonpolar regions. Subsequently, we characterized pH-jumps for saturated MCH solutions at different *x*_DMSO_ values. Similar to the case of water, higher mPAH solubility led to a proportional increase in pH-jumps upon illumination (Figure 4B, Figure S11). Thanks to enhanced solubility, the highest pH-jump in saturated conditions was reached in *x*_DMSO_ = 0.15 with 2.4 units, whereas in pure water, the pH-jump reached 1.5 units (Figure 4B).

Next, we evaluated the long-term light cycling stability (Figure 4C, Supporting Information S4) of a saturated photoacid solution (5.3 mM) in *x*_DMSO_ = 0.15—the sample for which the highest pH-jump is reached. Dissolving the photoacid in *x*_DMSO_ = 0.15 allows for significant improvements in stability: at least 2 weeks of stable light cycles is achieved with a lasting pH-jump that is higher than that in pure water (Figure 4C).

**Figure 5:**
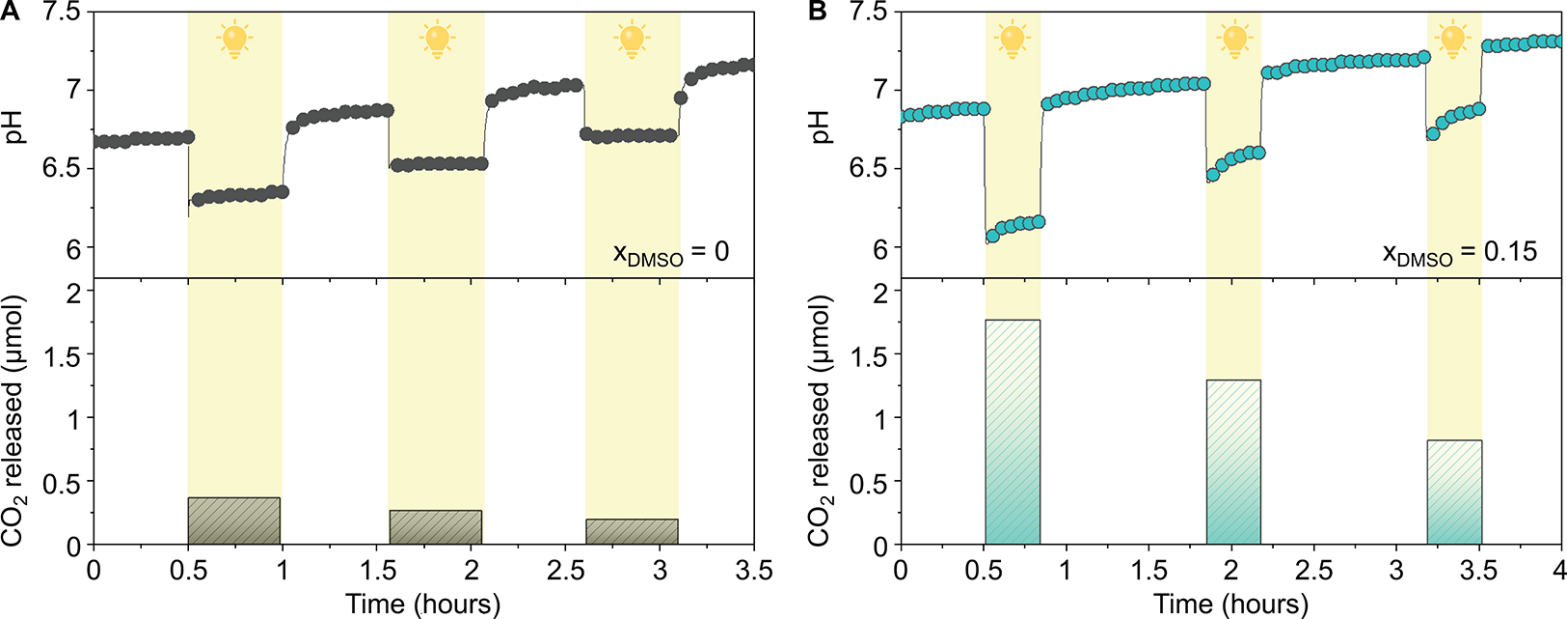
pH change (top) and cumulative CO_2_ release (bottom) from a solution with 0.2 mM mPA*H* and 0.2 mM KHCO_3_ in pure H_2_O (A), and 5.3 mM mPA*H* and 5.3 mM KHCO_3_ in *x*_DMSO_ = 0.15 (B) while cycling between light (yellow highlight) and dark.

Finally, as a proof-of-concept, we demonstrate the feasibility of using MCH in water–DMSO mixtures for light-driven CO_2_ capture–release (Figure 5). For this, CO_2_ was introduced by adding KHCO_3_ to the mixture at the ratio KHCO_3_:MCH = 1:1 to mitigate the buffering effect of KHCO_3_ (Supporting Information S6). As a result, a pH-jump could trigger a release of CO_2_. Because the DMSO cosolvent enables higher concentrations of the dissolved photoacid (0.2 mM in water vs 5.3 mM in *x*_DMSO_ = 0.15), we could use a higher KHCO_3_ concentration (given the aforementioned 1:1 ratio). Upon illumination, five times more CO_2_ was released in the water–DMSO mixture compared to water. After each consecutive illumination cycle, the amount of released CO_2_ gradually decreases (Figure 5). Given the enhanced stability and solubility of the merocyanine photoacid in water–DMSO mixtures (Figure 4), this demonstration serves as a steppingstone for enabling a stable light-driven CO_2_ capture–release.

## Conclusions

We show that enhanced stability and solubility of photoacids can be achieved when a simple water–DMSO mixture is used as a solvent. Up to *x*_DMSO_ = 0.25, the solution containing merocyanine returns to its initial pH within 1 h after illumination. We show that the chemical stability of the photoacid in water-based solutions containing DMSO can be extended by a factor of 20 (compared to pure water) under light cycling conditions. At *x*_DMSO_ = 0.15, a reversible pH-jump of up to 2.4 units can be achieved due to an enhanced solubility of merocyanine in the presence of DMSO. Using molecular dynamics simulations, we explain these results by the changes in the solvation environment of the photoacid. Our work shows that the use of binary solvent mixtures such as water–DMSO offers a simple way to optimize the pH-jump, solubility, and (photo)chemical stability of photoacids. We anticipate that the stability of other photoacids that are susceptible to hydrolysis can be dramatically improved in binary solvent mixtures due to their preferential solvation by aprotic solvent molecules. Finally, our work demonstrates that higher amounts of light-driven CO_2_ capture-release (relative to water) can be achieved for photoacids stabilized through the right solvation environment.

## Methods

### General

All chemicals were purchased from Sigma-Aldrich, TCI Chemicals, VWR International or Chemie Brunschwig AG and used without further purification. To avoid variations in degree of degradation, solutions were always freshly prepared before experiments and/or analysis unless noted otherwise. UV–vis absorption spectra were collected using an Agilent Cary 5000 or PerkinElmer Lambda 35. The spectra were measured in 70 μL UV-microcuvettes (BRAND), unless specified otherwise. The pH was measured using Mettler Toledo pH electrodes LE438 (polyester junction, 12 mm diameter) or LE422 (ceramic junction, 4.3 mm diameter). pH data was recorded using an Oakton pH 700 benchtop meter and logged every 3 s by the CyberComm 2700 computer software. Nuclear magnetic resonance (NMR) spectra were measured on a Bruker ASCEND 400 MHz spectrometer. For NMR measurements, D_2_O (99.9%, Chemie Brunschwig AG) or DMSO-*d*_6_ (99.9%, Sigma-Aldrich) solvents were used for sample preparation. Chemical shifts were referenced to the residual ^1^H signals of the solvent. In pH-jump measurements and photochemical degradation studies, photoacids were illuminated using a 405 nm LED (M405L4, 14 mW/cm^2^, Thorlabs), controlled by an LED driver (LEDD1B, Thorlabs). Light modulation cycles were programmed by using a waveform generator (DG1022z, Rigol).

### Synthesis

Synthesis and characterization are provided in the Supporting Information S1.

### pH-Jump and Reversibility

To ensure that all solutions have a similar starting pH of 5.25 ± 0.2, 2 vials containing basic and acidic solutions of the photoacid were prepared in the respective solvent mixture (described below). First, appropriate amounts of the photoacid were dissolved in DMSO (99.9%, VWR International AG). Next, to each vial was added an aqueous solution of 10 mM NaOH or HCl to achieve the targeted water–DMSO ratio. To ensure an optimal response of the pH electrode, 20 mM NaCl was added to the photoacid solutions. Finally, acidic and basic solutions were titrated to reach the target pH. To avoid light exposure, solutions were always kept in polypropylene vials (CELLSTAR Greiner Bio-One), wrapped in aluminum foil. The pH-jump and reversibility were measured in a 2 cm × 2 cm × 2 cm quartz cuvette (Portmann Instruments). For each measurement, 6 mL of photoacid solution was used and continuously stirred with a 5 mm Teflon stirring bar, rotating at 200 rpm. The cuvette was placed in a custom-made optical cell (Supporting Information S2, Figure S3), which had an 18 × 18 mm window for light exposure of 1 or 5 min (405 nm LED, M405L4, 14 mW/cm2, Thorlabs) and an airtight lid holding the pH electrode.

Prior to each experiment, the pH electrodes were calibrated by a three-point calibration with technical buffer standards (pH 4, 7, 10). To minimize the drift effect due to different solvent compositions inside the pH electrode and measured liquid, prior to each measurement, the pH electrode was stabilized in the respective solvent mixture for 20 min. It is important to note that while with such a pH measurement approach we followed the manufacturer recommendation of measurements in organic solvents,^73^ the obtained results do not account for possible differences of the liquid junction potentials (LJP). LJP can be different in solvent mixtures compared to pure water^66,67^ and therefore can affect the readings of the pH meter.^68^ Therefore, the pH values in different solvent mixtures should be treated as trends rather than as absolute values.

### Chemical Stability

To study the chemical stability of MCH in different solvents, we prepared the following solutions: 0.2 mM in D_2_O, 5.3 mM in *x*_DMSO-*d*6_ = 0.15 and 17.5 mM in pure DMSO-*d*_6_. The samples were measured using amber NMR tubes (5 mm, Norell) that prior to each use were cleaned with water, acetone, and deuterated solvent and dried overnight at elevated temperature. Assignment of the characteristic peaks (a, b′, c, c′) was done in accordance with a prior study by Berton et al.^13^ We tracked the degradation of the photoacid samples using positions of the (CH_3_)_2_ singlet NMR peaks, denoted as c, c′ or c* for MCH/MC^−^, SP and indolium moiety (D2), respectively (Figure 2, Supplementary Figure S5). We also measured a reference spectrum of salicylaldehyde (D1) in D_2_O to reference the position of the d*. All data was processed and analyzed using the MestReNova software. We monitored the samples over 12 weeks.

### Photochemical Stability

To evaluate the photochemical stability, the photoacid solutions were subjected to the following light modulation cycles using a 405 nm LED (*M405L4*, 14 mW/cm^2^, Thorlabs): 1 min light on and 58 min light off for dilute samples (Figure 3A). To calculate the degradation percentage, we compared the released effective proton concentration estimated from the pH-jump with cycling pH (pH_*t*_*x*__) to the amount measured for the first cycle (pH_*t*_0__) (eq 2). The half-life *t*_50%_ is the time at which 50% of degradation is reached (Figure 3C). In other words, *t*_50%_ is the time at which only half of the protons is released during pH-jump compared to the first cycle (eq 3).
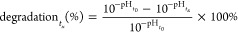
2
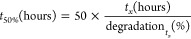
3

To compare the chemical and photochemical degradations, an additional 6 mL of each solution was stored in a vial in the dark for the duration of the light cycle experiment. At the end of the light cycle measurements, we performed a single pH-jump measurement of this sample (Supplementary Figures S5 and S3C).

### Solubility

Due to different solvation properties of water and DMSO of the merocyanine molecule, we studied whether different solubilities can be achieved depending on the preparation procedure. The step-by-step procedure is provided in Supporting Information S5.

### Photo-Driven CO_2_ Release

The procedure of CO_2_ release experiments is provided in the Supporting Information S6.

### Simulation Methodology

Molecular dynamics simulations of periodic cubic boxes were performed in OpenMM software.^74^ Each system contained one solute molecule (merocyanine or spiropyran) placed at the center of a box surrounded by a sufficient quantity of solvent to approximate infinite dilution. The total number of solvent molecules was set to 2000, and the molar fractions of DMSO, *x*_DMSO_ = 0, 0.05, 0.15. 0.3, and 1 were considered. Initial configurations were generated using PACKMOL,^75^ and the input files were prepared with fftool^76^ and polarizer^77^ utilities. A cutoff of 12 Å was set for nonbonded interactions, with the tail correction applied for energy and pressure. The particle mesh Ewald (PME) method was used to evaluate electrostatic energies, with a tolerance of 10^–5^. Bonds involving hydrogen atoms and the H–O–H angle of water were constrained using the SHAKE algorithm. A time step of 1 fs was considered. A dual Langevin thermostat and Monte Carlo barostat were applied for temperature and pressure control, with the values set to 298 K and 1 bar, respectively. The systems were equilibrated for 5 ns in the NPT ensemble, and then, 100 ns trajectories were generated in the NVT ensemble.

Drude induced dipoles were used to represent explicit polarization effects.^78^ Each site consisted of a positively charged Drude core (DC) with a negatively charged Drude particle (DP) of a mass of 0.4 au attached to the core with a harmonic spring (*k*_D_ = 4184 kJ/mol). The maximum elongation of the spring was constrained to 0.05 Å in order to prevent a “polarization catastrophe”.^79^ Partial charges of the DPs were evaluated from atomic polarizabilities, . Only nonhydrogen atoms were treated as polarizable, with the atomic polarizability of hydrogen atoms summed onto the polarizability of the atoms to which they are attached. Thole damping function^80^ with the universal parameter of 2.6^81^ was used to smear short-range interactions between the induced dipoles. The relative motion of DPs with respect to their cores was regulated at a temperature of 1 K as an approximation of the self-consistent regime.^78^

Water (W) was modeled using the existing SWM4-NDP polarizable force field.^82^ Polarizable models of merocyanine (MCH) and spiropyran (SP) were developed following the CL&Pol approach,^83,84^ while the force field for DMSO was already published earlier.^83^ The atomic partial charges of the solutes (Table S4) were evaluated using the CHelpG methodology^85^ at the MP2/cc-pVTZ level at previously optimized geometries in Gaussian.^86^ Bonded and nonbonded Lennard–Jones interaction parameters were taken from the OPLS-AA force field.^87^ Atomic polarizabilities were adapted from the work of Schröder.^88^ Initial well-depths (ε_ij_) of Lennard–Jones potential were modified in order to remove double counting of polarization effects,^83^ already represented by Drude-induced dipoles. Water–water interaction parameters were not modified and the interactions of water with other species were scaled only partially since the water model was already polarizable.^84^ Following the assumption of infinite dilution, solute–solute interactions were not modified either. All of the molecules/ions were considered as entire fragments. The scaling factors (*k*_*ij*_), evaluated using the predictive scheme,^83^ are given in Table S5. The atomic diameters (σ) were kept unchanged.

TRAVIS^89,90^ software was used for the structure analysis of the trajectories, and the spatial distribution functions (SDF) were visualized in VMD.^91^ The reference *xy* plane passes through C2, N1, and C3 (atomic labels provided in Figure S14) atoms for SDFs of SP, and through C10, C11, and C12 atoms for SDFs around the double C=C bond of MCH.

## Supporting Information Available

Synthetic and experimental procedures, supplementary calculations, and simulation methodology (PDF)
