# Supplementary material for: Solvation-Tuned Photoacid as a Stable Light-Driven pH Switch for CO2 Capture and Release
Source: Chem Mater. 2023 Dec 20;36(3):1308–17. doi: 10.1021/acs.chemmater.3c02435 (PMC10877570; doi:10.1021/acs.chemmater.3c02435)
Supplement: Supplementary file 1 — cm3c02435_si_001.pdf [file cm3c02435_si_001.pdf]

SUPPLEMENTARY INFORMATION

Solvation-tuned Photoacid as Stable Light-Driven pH Switch for CO_2 Capture and Release

Anna de Vries[1], Kateryna Goloviznina[2], Manuel Reiter[1], Mathieu Salanne[2,3], Maria R. Lukatskaya[1*]

[1] Electrochemical Energy Systems Laboratory, Department of Mechanical and Process Engineering, ETH Zurich, 8092 Zurich, Switzerland
[2] Sorbonne Université, CNRS, Physico-Chimie des Électrolytes et Nanosystèmes Interfaciaux, PHENIX, F-75005 Paris, France
[3] Institut Universitaire de France (IUF), 75231 Paris, France

Corresponding Author:
* mlukatskaya@ethz.ch

Table of contents

1. **Chemical synthesis** 3
    - i. Procedure
    - ii. Analytical data
2. **pH-jump and reversibility** 4
    - i. Calculation of reversibility time
    - ii. Custom photo-switching setup
3. **pK_a determination** 6
    - i. Procedure
    - ii. UV-Vis absorption results and fitting
4. **Stability** 7
    - a. Chemical degradation by NMR
    - b. Photochemical degradation by light cycles
        - i. pH-jump
        - ii. Thin layer configuration
5. **Solubility** 10
    - i. Procedure
    - ii. UV-Vis absorption calibration curves and solubility result
    - iii. Higher pH-jump with higher solubility
6. **Photochemical CO_2 release detection** 14
    - i. Procedure
    - ii. Calibration
    - iii. Data
7. **Simulation Methodology** 16

1. Chemical Synthesis of merocyanine

i. Procedure

The synthesis of the starting compound and merocyanine followed the previously reported procedures.[S1,2] In brief, the starting compound, 2,3,3-trimethyl-1-(3-sulfonatepropyl)-3H-indolium (A1), was prepared by adding 1,3-propanesultone (2 mL, 22.7 mmol, >99%, TCI Chemicals) and 2,3,3-trimethylindolenine (3 mL, 19.1 mmol, ≥97%, TCI Chemicals) to 12 mL acetonitrile (99%, Sigma-Aldrich). The mixture was deaerated by bubbling N_2 for 1 hour, then stirred at 80 °C for 10 hours. The pale violet solid was washed with ethyl acetate (99.7%, Sigma-Aldrich) and collected after vacuum filtration (2.1 g, 7.4 mmol, 39% yield). Finally, merocyanine (MCH) was prepared by adding A1 (2 g, 7.11 mmol) and salicylaldehyde (0.96 mL, 9.16 mmol, ≥99%, Sigma-Aldrich) to ethanol (44 mL, extra dry over molecular sieve, 99.5%, Fischer Scientific AG). The mixture was then stirred under N_2 and refluxed (78 °C) overnight. The formed merocyanine (orange solid) was then washed with ethyl acetate (3 times with 22 mL) and collected by vacuum filtration (2.0 g, 5.2 mmol, 74% yield). The synthesized merocyanine was stored in an amber vial (wrapped in aluminum foil) in a desiccator at room temperature.

ii. Analytical data

Synthesized MCH was characterized by ^{1}H-NMR and ^{13}C-NMR (Figure S1): ^{1}H NMR (400 MHz, DMSO) δ 8.61 (d, *J* = 16.4 Hz, 1H), 8.29 (dd, *J* = 8.0, 1.6 Hz, 1H), 8.07 – 7.98 (m, 1H), 7.94 – 7.82 (m, 2H), 7.69 – 7.57 (m, 2H), 7.48 (ddd, *J* = 8.5, 7.1, 1.6 Hz, 1H), 7.08 – 6.95 (m, 2H), 4.82 (t, *J* = 7.9 Hz, 2H), 2.66 (t, *J* = 6.4 Hz, 2H), 2.25 – 2.13 (m, 2H), 1.78 (s, 5H). ^{13}C NMR (101 MHz, DMSO) δ 181.75, 158.99, 148.68, 143.46, 140.91, 135.71, 129.77, 129.12, 122.94, 121.33, 120.04, 116.60, 115.06, 111.45, 51.88, 47.31, 30.67, 26.41, 24.57.

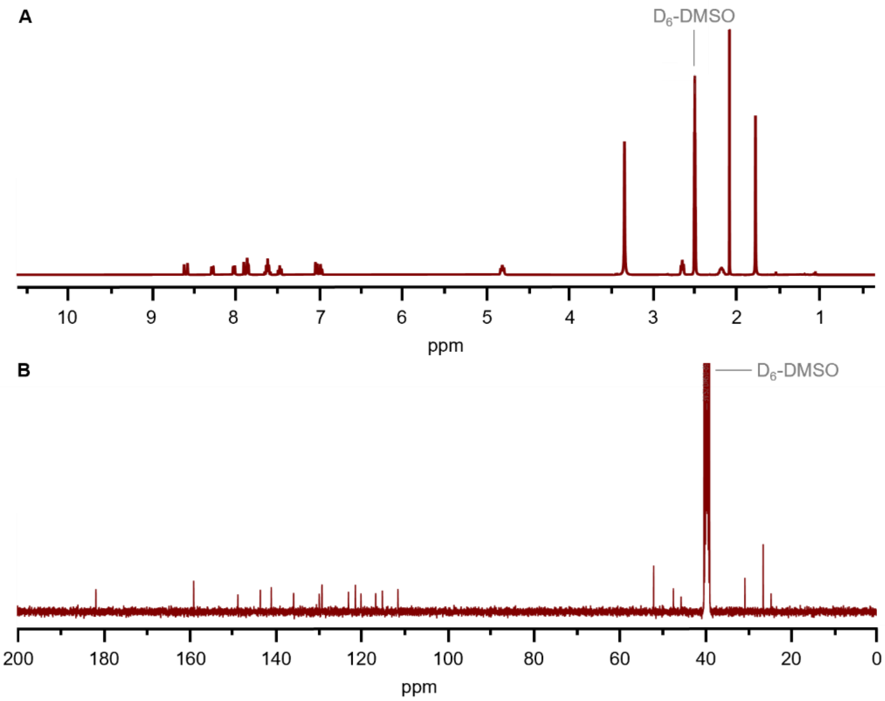

Figure S1. (A) ^{1}H-NMR and **(B)** ^{13}C-NMR spectrum of synthesized merocyanine photoacid in d_6-DMSO.

2. pH-jump and reversibility

i. Calculation of reversibility time

The reversibility time was estimated using extrapolation of the linear thermal relaxation region (first 10 minutes in the dark) to pH = pH^{GS} (Figure S2, Table S1, Equations S1-S2).

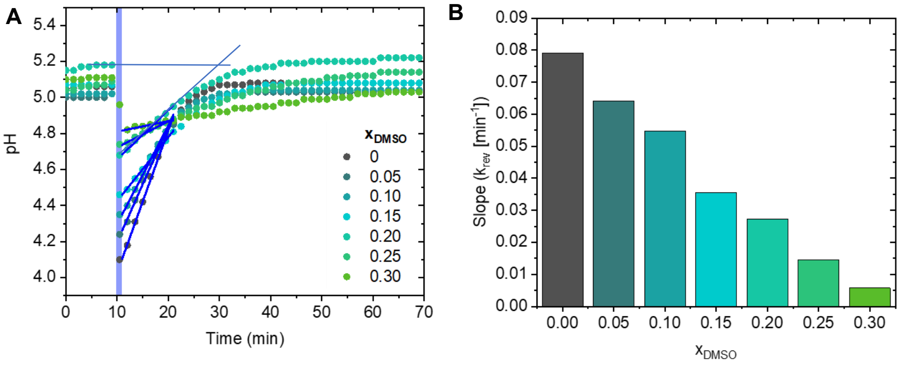

Figure S2. (A) Linear fit of the initial 10 minutes of MCH relaxation in the dark and **(B)** reversibility rate constant as a function of x_{DMSO} assuming first order (linear) kinetics.

Table S1. Intercept and slope corresponding to linear fit (Figure S2) to calculate t_{rev} (Equation S5).

X_{DMSO}	Intercept, b	Slope, a (k_{rev}) [min^{-1}]	pH^{GS}
**0**	3.23	0.079	5.06
**0.05**	3.55	0.064	5.03
**0.1**	3.75	0.055	5.04
**0.15**	4.07	0.036	5.08
**0.2**	4.38	0.027	5.22
**0.25**	4.58	0.015	5.14
**0.3**	4.75	0.006	5.11

$$pH_{rev} = a * t + b \qquad \textit{Equation S1}$$

$$t = t_{rev} \text{ when } pH_{rev} = pH^{GS}, \quad t_{rev} = \frac{pH^{GS} - b}{a} \qquad \textit{Equation S2}$$

ii. Custom photo-switching setup

A custom-made optical cell was designed for photo-switching experiments (Figure S3). The cell comprises of a cuvette holder (black polyoxymethylene, POM), that contains a 2 x 2 x 2 cm quartz glass cuvette, 3 removable shutters (black slides) and optional gas in- and outlet (red fittings). The cell is closed by a lid (beige polyether ether keton, PEEK) that has an inlet for the pH electrode (black shaft), which is held and sealed by compression of an o-ring (top lid). The photo-switching setup is contained in a black box that is covered with tin foil to avoid any light exposure other than LED light.

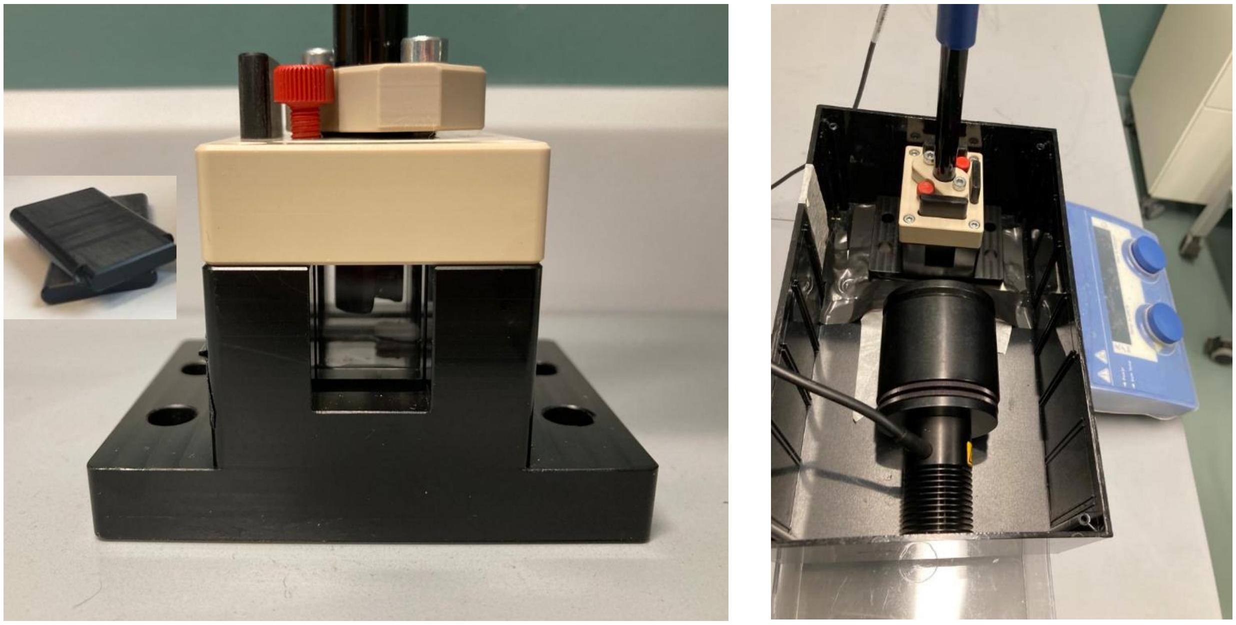

Figure S3. Custom-made optical cell (left), designed for photo-switching experiments. Photo-switching setup with optical cell, pH-meter, LED light and magnetic stirrer (right).

3. pK_a determination

The acidity of a molecule, given by its pK_a, can change based on the properties of its immediate environment, such as the polarity and basicity of the solvent.[S3,S4] Therefore, using UV-Vis titration we studied how the pK_a^{GS} of a mPAH changes in water-DMSO solvent mixture compared to pure water. Our experiments showed that the pK_a^{GS} of MCH decreases slightly from 6.

2 in pure water to 6.0, as calculated in a mixture with x_{DMSO}= 0.15 (Figure S4). As the change in pK_a is relatively small, we assume that the ratio MCH/MC^- does not change considerably across the solutions studied, when starting with the same pH. However, further studies in other water-DMSO ratios are needed to fully understand the relationship between solvent structure and the related acidity of photoacids.

i. Procedure

For the determination of the pK_a, the acidic and basic solutions of photoacid were prepared in a similar manner to pH-jump studies, arriving at the following compositions:

- Basic solutions: 0.08 mM mPAH + 10 mM NaOH + 20 mM NaCl;
- Acidic solutions: 0.08 mM mPAH + 10 mM HCl + 20 mM NaCl.

Next, we performed a pH titration by adding the basic solution to the acidic one to modify the pH from 3 – 9 with increments of 0.5. The UV-Vis absorption spectrum of a sample at each pH increment was measured. All samples were prepared and measured within 5 hours, for which a limited extent of degradation can be assumed[S5].

ii. UV-Vis absorption results and fitting

The pK_a was determined by analyzing the ratio between the absorption maximum of the protonated (A_{max}^{MCH}) and deprotonated peaks ($A_{max}^{MC^-}$ and A_{max}^{SP}) as a function of pH (Equation S3-S4). The pK_a was assigned the pH value at which the fraction PAH/PA^- is 0.5 (Figure S4C).

$$PAH = \frac{A_{max}^{MCH}}{A_{max}^{MCH}+A_{max}^{MC^-}+A_{max}^{SP}} \qquad \textit{Equation S3}$$

$$PA^- = \frac{A_{max}^{MC^-}+A_{max}^{SP}}{A_{max}^{MCH}+A_{max}^{MC^-}+A_{max}^{SP}} \qquad \textit{Equation S4}$$

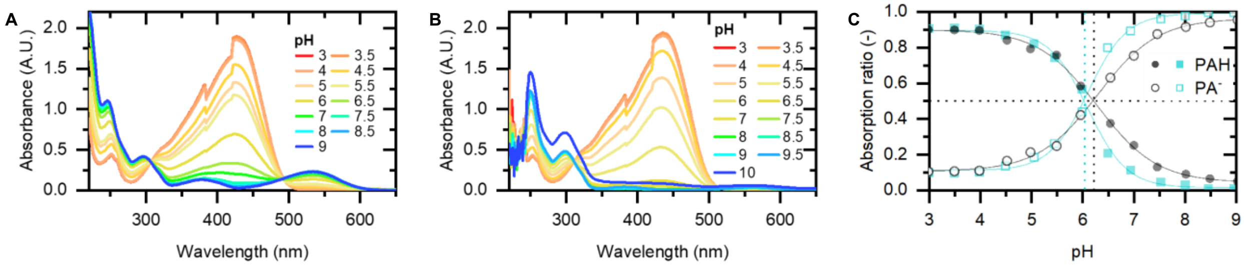

Figure S4. pH titration of PAH in **(A)** H_2O and **(B)** x_{DMSO} = 0.15, spectra increment with pH units of 0.5. **(C)** Absorption ratio between the absorption maximum (A_{max}) of MCH at 423nm, and MC- and SP peaks at 560 and 300 nm, respectively, of 0.08mM PAH in 20mM NaCl, with pH adjusted by mixtures of 10mM NaOH and 10mM HCl in water (black) and 15% DMSO (blue).

4. Stability

a. Chemical degradation by NMR

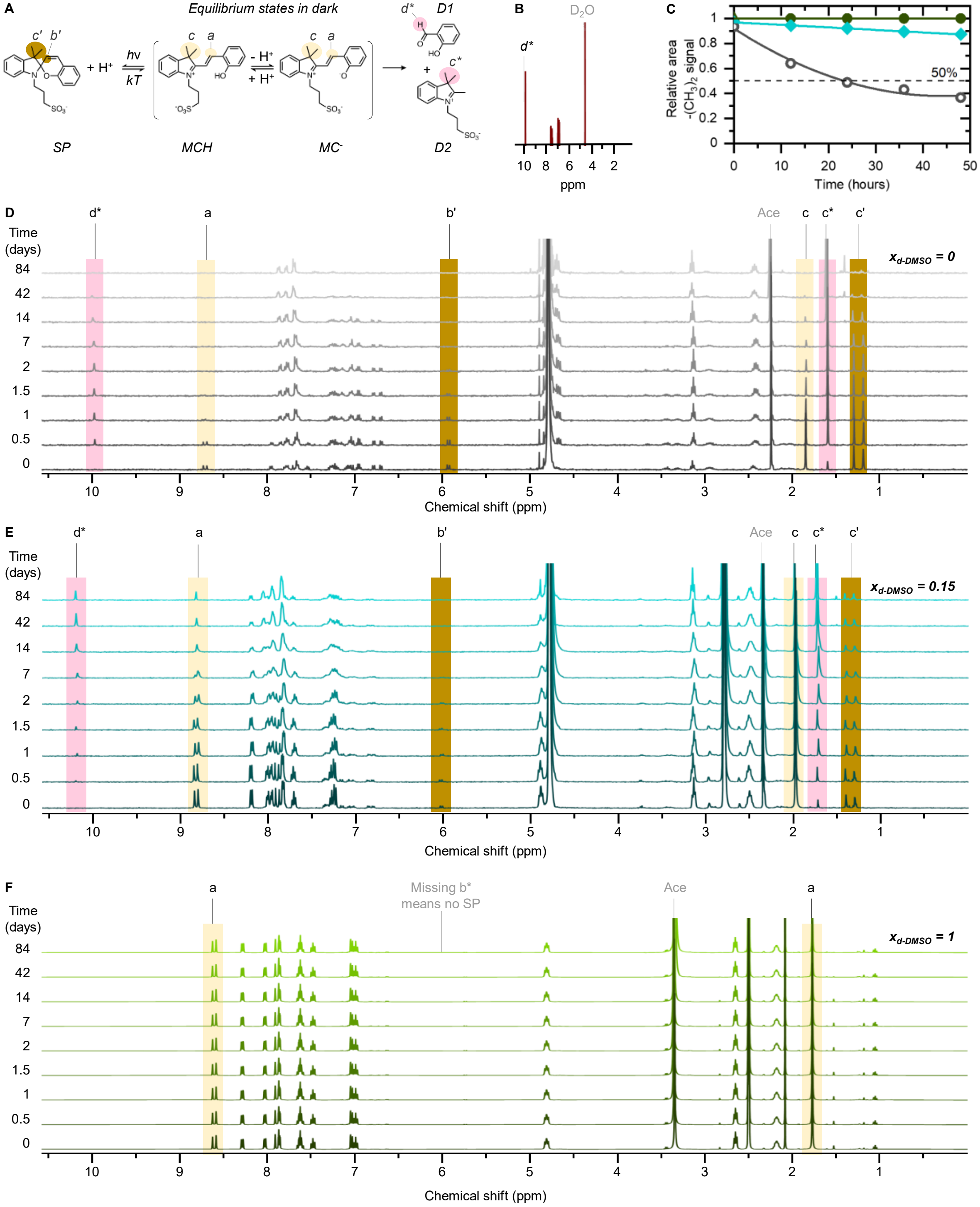

Figure 5. (A) Schematic of equilibrium between ring closed spiropyran (SP), protonated- and deprotonated Merocyanine (MCH and MC- respectively), and degradation products (D1 and D2). **(B)** Reference ^{1}H-NMR spectrum of salicylaldehyde. **(C)** Chemical degradation of first 2 days from ^{1}H-NMR signals. **(D)** ^{1}H-NMR spectra of PAH in D_2O (0.2mM), **(E)** $x_{d6\text{-}DMSO}$ = 0.15 (5.3mM) and **(F)** pure d_6-DMSO (17.5mM).

b. Photochemical degradation by light cycles

i. pH-jump data

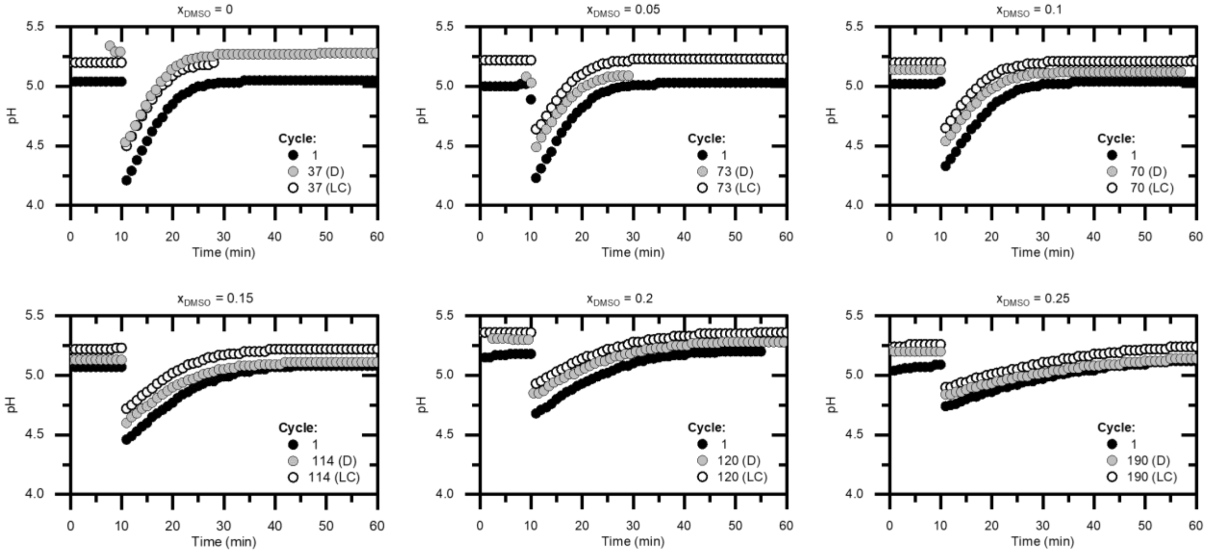

Figure S6. pH-jump of 0.08 mM MCH in different mole fractions of DMSO at the first light cycle, last light cycle (LC), and sample that remained in the dark at the same time of the last light cycle (D). Each cycle consists of 1 minute light and 58 minutes of darkness.

ii. Thin layer configuration

The measured pH-jump efficiency and reversibility time can be affected by lamp intensity, stirring efficiency, type of pH electrode, and light path length. Our measurements show that higher pH-jumps are observed in a cuvette with a path length of 5 mm compared to a cuvette with a 20 mm path length (Figure S7). Therefore, a thin layer configuration (5 mm), was used to determine the highest possible pH-jump. However, the pH electrode compatible with the small cuvette is not optimal for the long-term stability measurements, since the ceramic junction allows for the exchange of the filling solution with the photoacid solution (LE422, Mettler Toledo). Here, we again note that contributions and values of LJP for pH meter probes with different pH junctions types are not accounted for in current study, but can affect the readings of the pH meters. Therefore, measured pH values should be taken as approximate. For long-term stability measurements, the pH electrode with a gel electrolyte with minimal leakage was used (polyester junction, LE438, Mettler Toledo). However, due to the larger diameter of this pH electrode (12 mm), it was not compatible with the 5 mm path length cuvette. Consequently, long-term stability experiments were conducted in the larger cuvette, which do not allow for the highest achievable pH-jump.

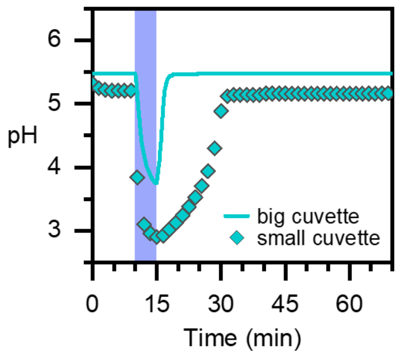

Figure S7. pH-jump of 5.3mM PAH in x_{DMSO} = 0.15 measured in 20 mm (big) or 5 mm (small) cuvette.

5. Solubility

i. Procedure

To study the solubility of merocyanine in water and DMSO, two types of solution series were evaluated:

- Pre-mix series: first, a solvent mixture with the desired water-DMSO ratio was prepared. Then, in a septum-sealed, amber vial, pre-mixed water-DMSO solvent was added incrementally to a known amount of photoacid using a glass syringe (10 µl, Hamilton). After each addition, the mixtures were sonicated for 20 minutes to ensure complete dissolution of the photoacid. This process was repeated until all photoacid was dissolved. The vials were left at room temperature (~22°C) for a minimum of 3 days to ensure that the photoacid remained dissolved.

- Post-mix series: here, the solubility was evaluated following two different protocols (described below).

*Incremental***:** first, a saturated solution of photoacid in pure DMSO was prepared (16.4 mM). Then, this solution was added to water in appropriate amounts to achieve the target water-DMSO ratio. If precipitation was observed upon addition, the process was repeated with a lower starting concentration in DMSO.

*UV-Vis***:** first, for each water-DMSO ratio, UV-Vis spectra of solutions with known concentrations between [0.01;0.06] mM were collected. Then, a calibration curve was obtained by plotting the absorption maximum as a function of the known photoacid concentration and its linear fitting (y=ax) (Figure S8). Second, a saturated solution of photoacid in pure DMSO was prepared by adding excess amount of photoacid to DMSO. Then, the supernatant was passed through a syringe filter (PTFE, 0.45 µm, 13 mm, Fisherbrand) and added to water to achieve the target water-DMSO ratio, if precipitation occurred the syringe filtration process was repeated. Third, the saturated solutions were diluted by a known amount of the corresponding solvent mixture, to reach the range between [0.01;0.06] mM (in the linear dynamic range of the UV-Vis absorption). Finally, from the absorption maxima of the diluted sample and the calibration curve, the exact concentration of the diluted sample was obtained. This value was then used together with the known dilution factor, to back-calculate the concentration of the saturated solution for each water-DMSO ratio.

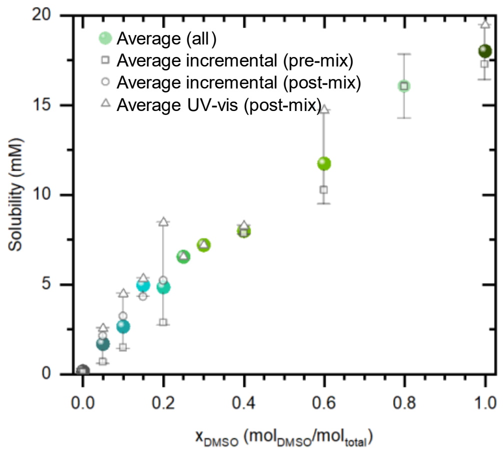

Figure S8. Solubility results of different methods: incremental (with pre- or post-mixed series) and UV-Vis (post-mixed). Average (all) are the same values as in Figure 4A.

ii. UV-Vis absorption calibration curves and solubility result

Calibration curves were obtained by linear fitting (y=ax) of the absorption maxima of the MCH peak (right panels in Figure S8). Fitting parameters are stated in table S2.

x_{DMSO} = 0

A = 20.3378 * C

S = 0.25965 ± 9.76872^{-4} mM

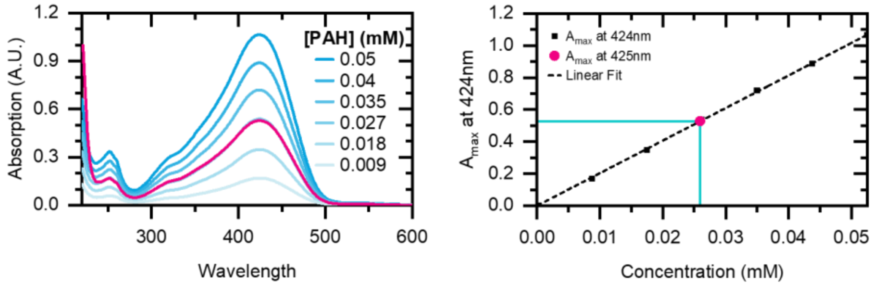

x_{DMSO} = 0.05

A = 21.70209 * C

S = 2.5427 ± 0.01255 mM

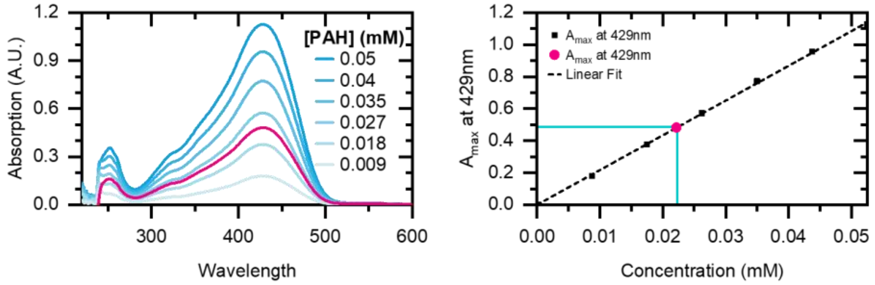

x_{DMSO} = 0.1

A = 20.55 * C

S = 4.4733 ± 0.0563

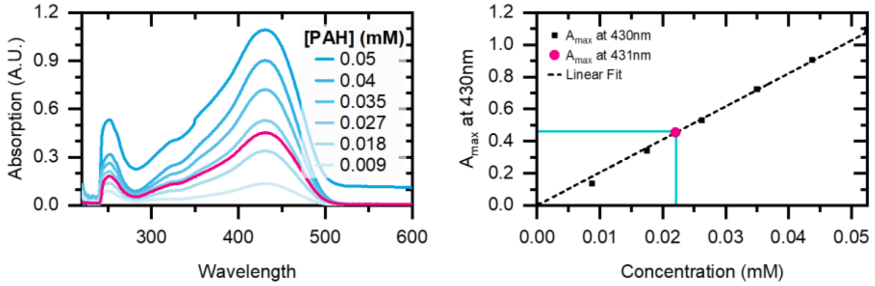

x_{DMSO} = 0.15

A = 20.43557 * C

S = 5.31654 ± 0.05532 mM

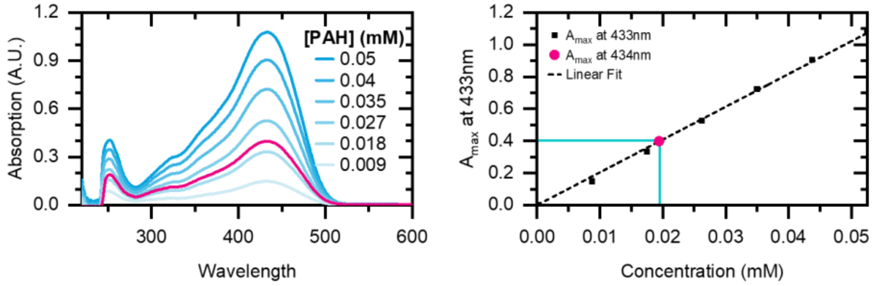

x_{DMSO} = 0.2

A = 15.638 * C

S = 3.37 ± 0.58 mM

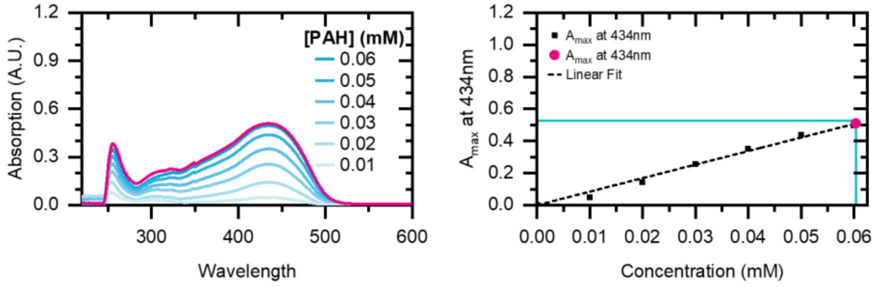

x_{DMSO} = 0.25

A = 15.7356 * C

S = 6.54724 ± 0.25659

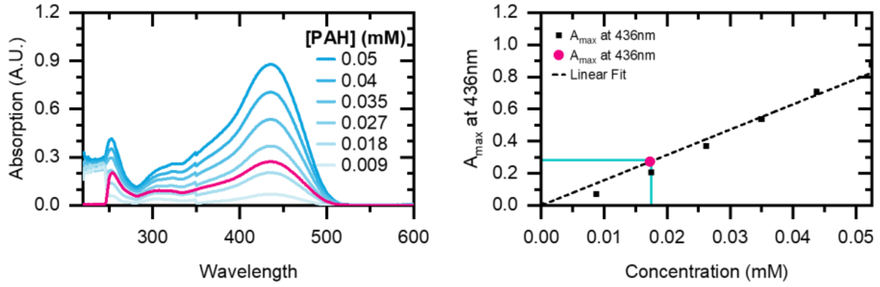

x_{DMSO} = 0.3

A = 14.76921 * C

S = 7.18984 ± 0.29893 mM

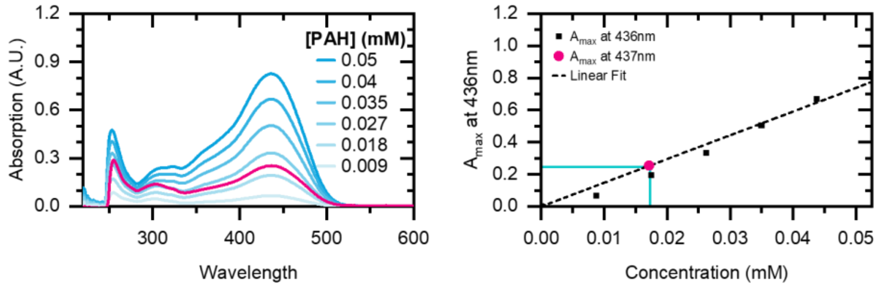

x_{DMSO} = 0.4

A = 15.66527 * C

S = 8.26152 ± 0.18411 mM

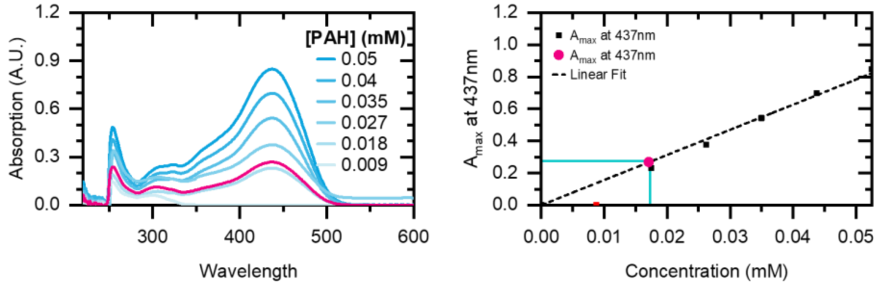

x_{DMSO} = 0.6

A = 19.81186 * C

S = 14.70954 ± 0.26519 mM

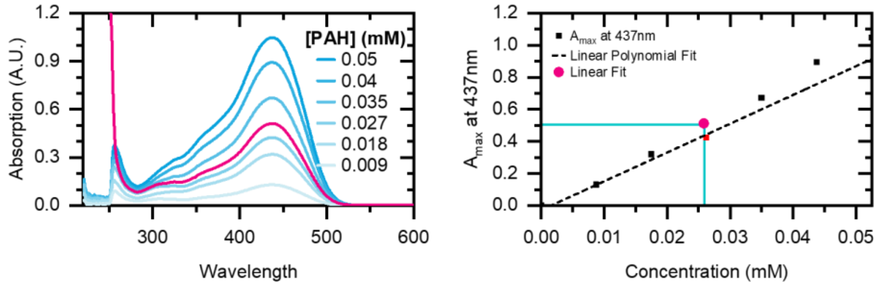

x_{DMSO} = 1.0

A = 19.97786 * C

S = 19.4735 ± 0.24143 mM

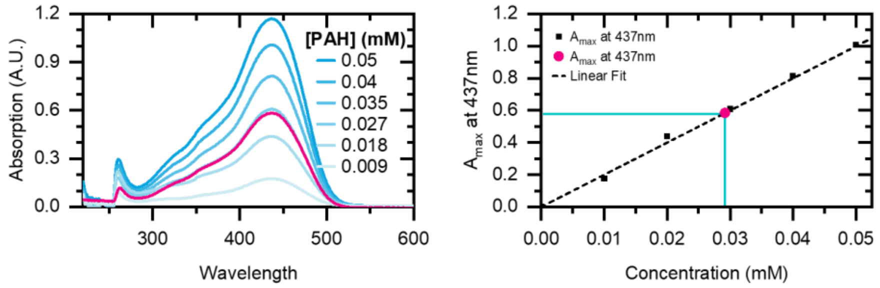

Figure S9. UV-Vis Absorption spectra (left) and calibration curve (right) of PAH dilutions (blue) and diluted saturated sample (pink)

Table S2. Fitting parameters from calibration curves and resulting solubility values.

x_{DMSO}	Slope calibration curve	R-square (COD)	Solubility (mM)
**0**	20.3378 ± 0.07623	0.99992	0.25965 ± 0.0010
**0.05**	21.70209 ± 0.10662	0.99986	2.5427 ± 0.0126
**0.1**	20.5484 ± 0.2554	0.99907	4.4733 ± 0.0563
**0.15**	20.43557 ± 0.21043	0.99936	5.31654 ± 0.05532
**0.2**	8.43835 ± 0.21701	0.99605	8.45257 ± 0.22311
**0.25**	15.73558 ± 0.59342	0.99154	6.54724 ± 0.25659
**0.3**	14.76921 ± 0.58954	0.99053	7.18984 ± 0.29893
**0.4**	15.66527 ± 0.34149	0.99763	8.26152 ± 0.18411
**0.6**	19.81186 ± 0.35085	0.99843	14.70954 ± 0.26519
**1**	19.97786 ± 0.24465	0.9991	19.4735 ± 0.24143

Table S3. MCH peak positions changing with DMSO mole fraction.

x_{DMSO}	MCH peak in calibration curves (nm)	MCH peak in sample (nm)
**0**	424	425
**0.05**	429	429
**0.1**	430	431
**0.15**	433	434
**0.2**	434	434
**0.25**	436	436
**0.3**	436	437
**0.4**	437	437
**1**	437	437

iii. Higher pH-jump with higher solubility

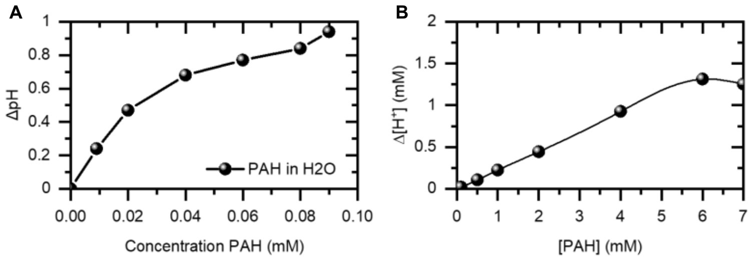

Figure S10. (A) pH-jump of MCH in water at different concentrations and **(B)** corresponding released proton concentration as a function of photoacid concentration.

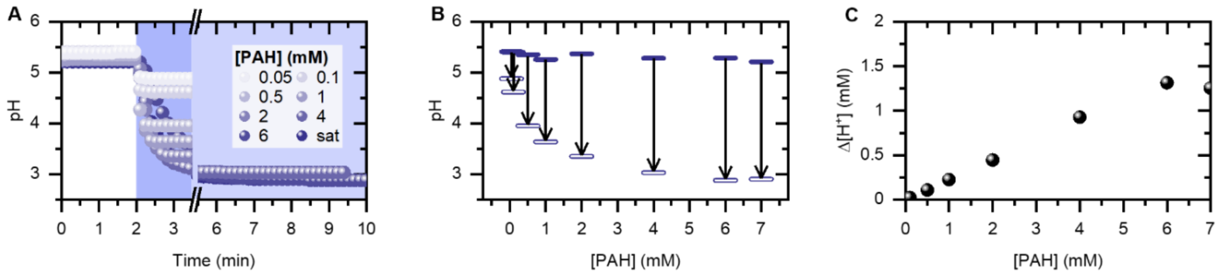

Figure S11. (A) pH-jump of MCH under continuous illumination in x_{DMSO} = 0.15 at different concentrations, **(B)** corresponding pH-jump and **(C)** released proton concentration as a function of photoacid concentration.

6. Photochemical CO_2 release detection

i. Procedure

CO_2 release experiments were carried out with 20 mL solutions of 0.2 mM MCH in water and 5.3 mM in x_{DMSO} = 0.15 with molar equivalents of $KHCO_3$. The solution was placed in a custom-made optical cell that contained gas in- and outlet valves, pH-meter, magnetic stirbar, window for LED light (5 cm diameter) and a CO_2 sensor (*SCD41*, Sensirion) in the headspace. The remaining headspace volume was 0.9 mL. At the beginning of each experiment, the headspace was flushed with 100 ml/min N_2 for 3 minutes. Next, the in-/outlet valves were closed and the pH and CO_2 sensors were left to stabilize for at least 30 minutes. To evaluate the photochemical release of CO_2 the solutions were subject to three cycles of: 30 minutes of light and 30 minutes of darkness in the case of water solvent, and 20 minutes of light and 60 minutes of darkness in the case of the water-DMSO mixture. After each cycle of light, the valves were opened and the headspace was purged with N_2 to avoid any re-absorption of prior released CO_2.

ii. Calibration curve

To quantify the CO_2 release, the CO_2 sensor was calibrated by purging different concentrations of CO_2/N_2 mixtures, controlled by mass flow controllers into the headspace of the optical cell (Figure S12A). The calibration curve was obtained by plotting the measured CO_2 concentration by the sensor (ppm_{sensor}) against the MFC input concentration (ppm_{MFC}) and applying a linear fit (Figure S12B, Equation S5).

$$ppm_{sensor} = -0.11164 + 5.855 * ppm_{MFC} \qquad \textit{Equation S5}$$

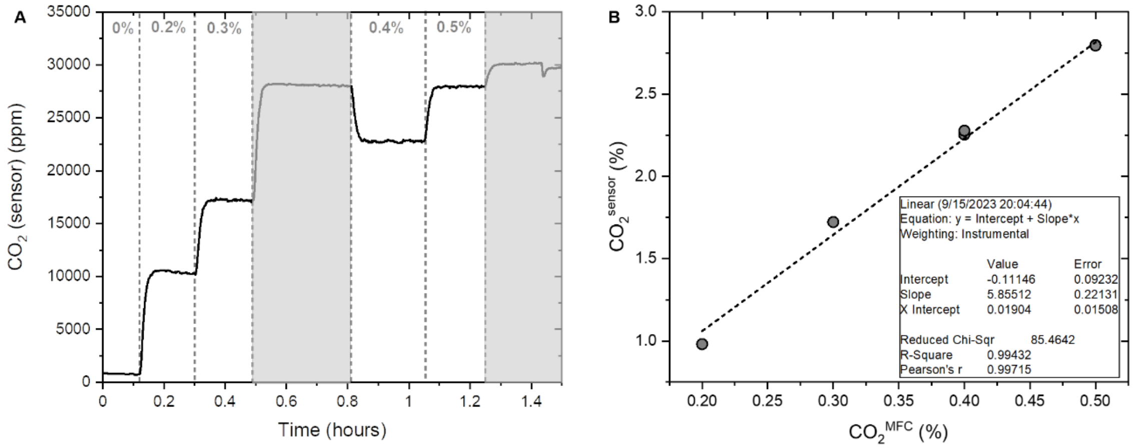

Figure S12. CO_2 sensor measurement while varying CO_2 concentration in N_2 (A) and corresponding calibration curve (B).

iii. Data

During experiments, CO_2 release was continuously detected by the CO_2 sensor in the headspace. The measured sensor data was first converted to calibrated data by solving equation S5 for ppm_{MFC} to obtain actual gas concentrations. Then, the resulting gas concentration was converted to molar values by multiplying the headspace volume (Equation S6) and finally solving the ideal gas law (Equation S7).

$$V_{CO_2} = ppm_{MFC} * V_{headspace} \qquad \textit{Equation S6}$$

$$n_{CO_2} = \frac{p * V_{CO_2}}{RT} \qquad \textit{Equation S7}$$

Where V_{CO2} is the actual measured CO_2 volume in m^3, $V_{headspace}$ (in m^3) corresponds to the 0.9 mL headspace volume of the opical cell, n_{CO2} is amount of mol of CO_2 measured in the headspace, p is the pressure in Pa ($1 * 10^5$ Pa), R the ideal gas constant (8.31 J K^{-1} mol^{-1}) and T the temperature (293 K).

Finally, cumulative CO_2 release values of each cycle (Figure 5) were determined by subtracting the measured concentration at the beginning of light, from the concentration at the end of light exposure (Figure S13).

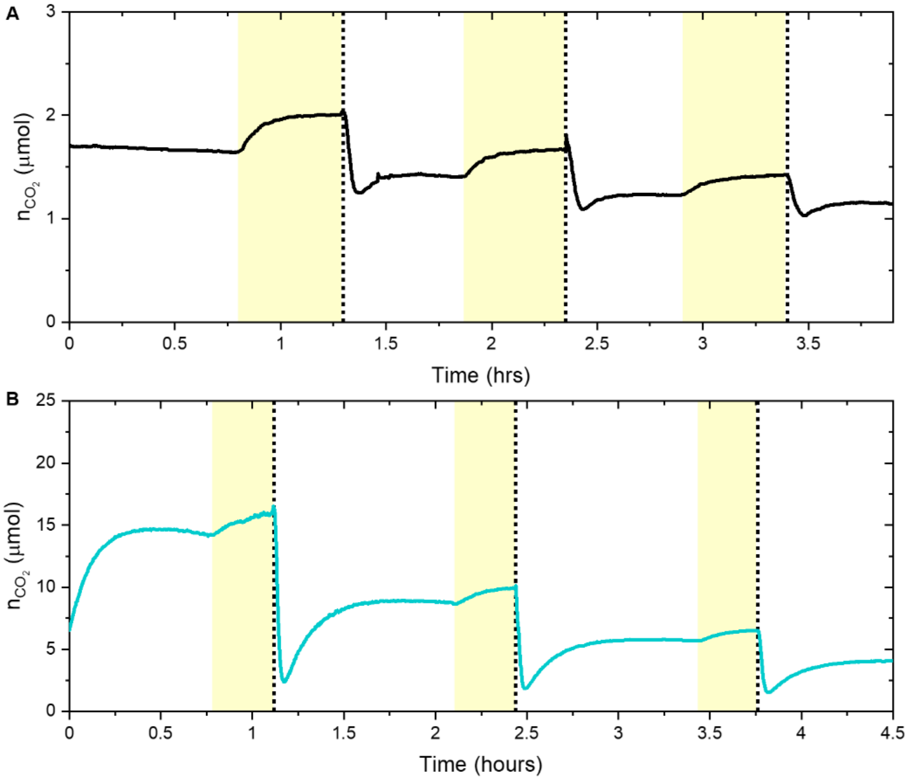

Figure S13. Amount of CO_2 detected in the headspace during 3 consecutive photochemical CO_2 release cycles of light (yellow highlight) from concentrated MCH and molar equivalent $KHCO_3$ in water (A) and in x_{DMSO} = 0.15 (B). An N_2 purge after each light cycle is indicated by dotted line.

7. Simulation methodology

Table S4. Force field parameters of merocyanine and spiropyran. The corresponding labels are given in Figure S14.

**Merocyanine (MCH)**					**Spiropyran (SP)**				
**Atom**	q	σ	ε [a]	α [b]	Atom	q	σ	ε [a]	α [b]
**N1**	-0.170	3.25	0.711	1.208	N1	-0.520	3.25	0.711	1.698
**C2**	0.200	3.55	0.293	1.277	C2	0.510	3.50	0.276	1.016
**C3**	0.335	3.50	0.276	1.016	C3	0.100	3.50	0.276	1.016
**C4**	-0.145	3.55	0.293	1.600	C4	-0.150	3.55	0.293	1.755
**C5**	-0.065	3.55	0.293	1.600	C5	-0.200	3.55	0.293	1.755
**C6**	-0.100	3.55	0.293	1.600	C6	-0.100	3.55	0.293	1.755
**C7**	-0.155	3.55	0.293	1.600	C7	-0.250	3.55	0.293	1.755
**C8**	0.165	3.55	0.293	1.277	C8	0.300	3.55	0.293	1.432
**C9**	-0.055	3.55	0.293	1.277	C9	-0.100	3.55	0.293	1.432
**C10**	-0.210	3.54	0.318	1.600	C10	-0.050	3.54	0.318	1.755
**C11**	-0.080	3.54	0.318	1.600	C11	-0.250	3.54	0.318	1.755
**C12**	0.065	3.55	0.293	1.277	C12	0.100	3.55	0.293	1.432
**C13**	0.360	3.55	0.293	1.277	C13	0.270	3.55	0.293	1.432
**C14**	-0.210	3.55	0.293	1.600	C14	-0.230	3.55	0.293	1.755
**C15**	-0.055	3.55	0.293	1.600	C15	-0.100	3.55	0.293	1.755
**C16**	-0.100	3.55	0.293	1.600	C16	-0.120	3.55	0.293	1.755
**C17**	-0.215	3.55	0.293	1.600	C17	-0.180	3.55	0.293	1.755
**C18**	-0.180	3.50	0.276	1.985	C18	-0.180	3.50	0.276	1.985
**C19**	-0.180	3.50	0.276	1.985	C19	-0.180	3.50	0.276	1.985
**C20**	0.040	3.50	0.276	1.662	C20	0.185	3.50	0.276	1.662
**C21**	0.285	3.50	0.276	1.662	C21	0.145	3.50	0.276	1.662
**C22**	-0.390	3.50	0.276	1.662	C22	-0.420	3.50	0.276	1.662
**SO3**	1.190	3.55	1.046	1.553	SO3	1.190	3.55	1.046	1.553
**OS3**	-0.680	3.15	0.837	1.144	OS3	-0.680	3.15	0.837	1.144
**HA**	0.100	2.42	0.126	0.000	HA	0.100	2.42	0.126	0.000
**H10-11**	0.100	2.42	0.126	0.000	H10-11	0.100	2.42	0.126	0.000
**HC**	0.060	2.50	0.126	0.000	HC	0.060	2.50	0.126	0.000
**H1N**	0.050	2.50	0.126	0.000	H1N	0.050	2.50	0.126	0.000
**HS3**	0.090	2.50	0.126	0.000	HS3	0.090	2.50	0.126	0.000
**H2N**	0.000	2.50	0.126	0.000	H2N	0.000	2.50	0.126	0.000
**OH**	-0.555	3.07	0.711	1.467	OA	-0.370	2.90	0.586	1.144
**HO**	0.430	0.00	0.000	0.000					

Units are: q / e^-, σ / Å, ε / kJ mol^{-1}, α / $Å^3$.

[a] Well-depth values of Lennard-Jones potential are provided before scaling.

[b] Atomic polarizability of hydrogen atoms was merged onto the polarizability of the atoms to which they are bonded.

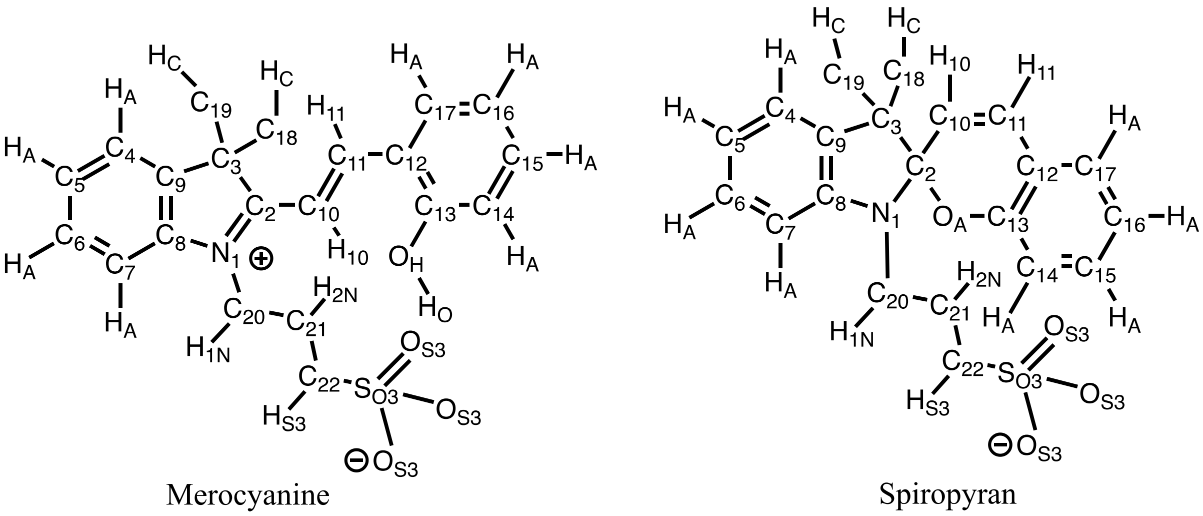

Figure S14. Structural formulae of merocyanine and spiropyran

Table S5. Scaling coefficients for the modification of non-bonded attractive interactions

Fragment *i*	Fragment *j*	k_{ij}	Fragment *i*	Fragment *j*	k_{ij}
MCH	DMSO	0.44	SP	DMSO	0.38
MCH	W	0.71	SP	W	0.71
DMSO	DMSO	0.68	DMSO	W	0.71

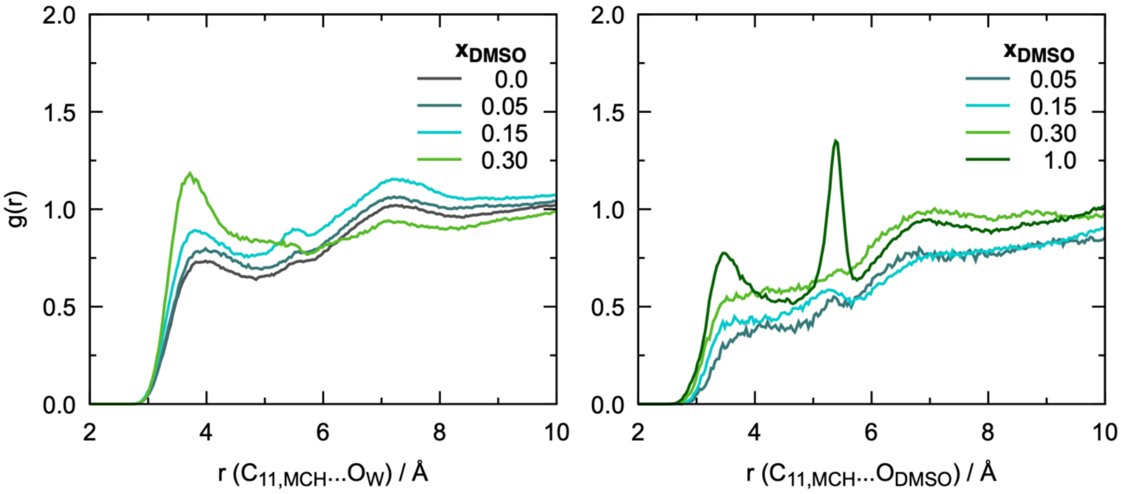

Figure S15. Radial distribution functions of oxygen atoms of water (left) and DMSO (right) around carbon atom of the double bond of merocyanine.

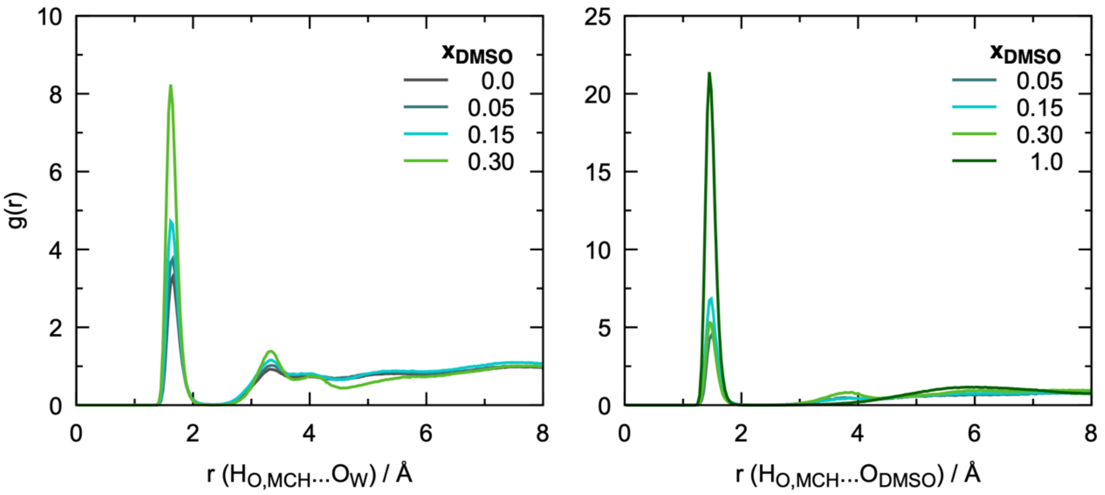

Figure S16. Radial distribution functions of oxygen atoms of water (left) and DMSO (right) around hydrogen atom of hydroxyl group of merocyanine.

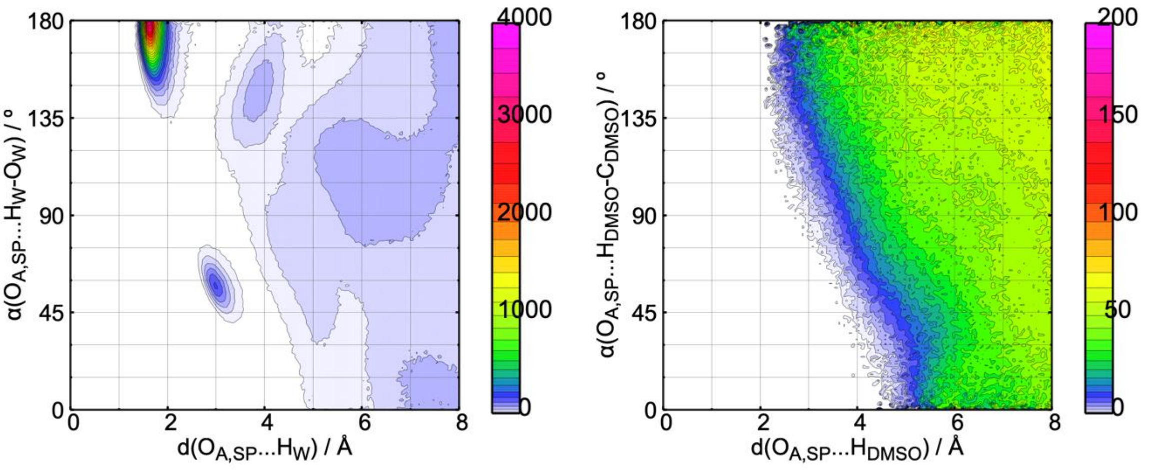

Figure S17. Probability contours revealing hydrogen bonds of SP with water (left) and DMSO (right) in a system with x_{DMSO} = 0.15. The x-axes represent the distances between the hydrogen atoms of a solvent and the acceptor oxygen atom of the solute (A). The y-axis represents the angles formed by the D-H...A hydrogen bonds, where D is a donor atom attached to the hydrogen.

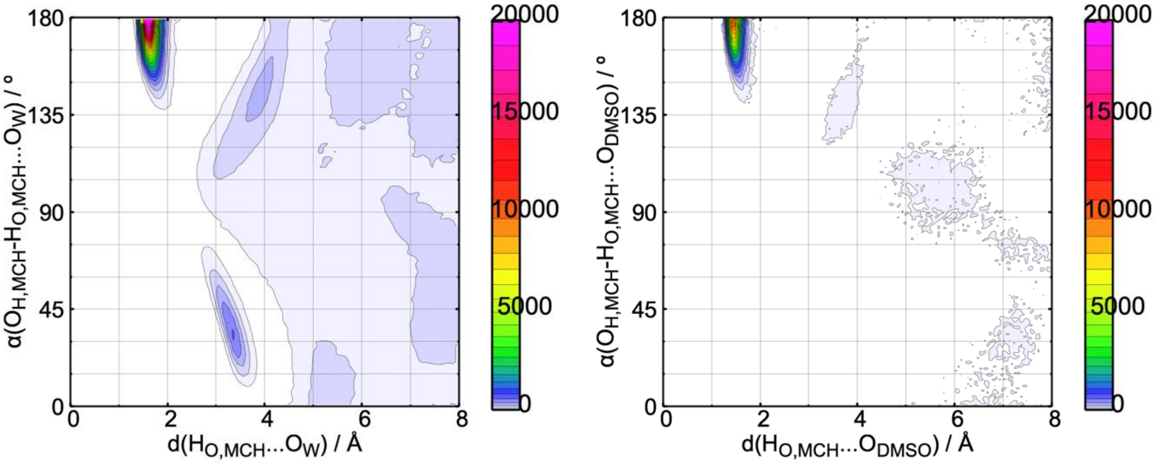

Figure S18. Probability contours revealing hydrogen bonds of MCH with water (left) and DMSO (right) in a system with x_{DMSO} = 0.15. The x-axes represent the distances between the hydrogen atom of the solute and the acceptor oxygen atoms of a solvent (A). The y-axis represents the angles formed by the D-H...A hydrogen bonds, where D is a donor atom attached to the hydrogen.

Supplementary references

S1. Shi, Z., Peng, P., Strohecker, D., and Liao, Y. (2011). Long-lived photoacid based upon a photochromic reaction. J. Am. Chem. Soc. *133*, 14699-14703. 10.1021/ja203851c.

S2. Liu, J., Tang, W., Sheng, L., Du, Z., Zhang, T., Su, X., and Zhang, S.X. (2019). Effects of Substituents on Metastable-State Photoacids: Design, Synthesis, and Evaluation of their Photochemical Properties. Chem. Asian J. *14*, 438-445. 10.1002/asia.201801687.

S3. Pines, D., and Pines, E. (2006). Solvent Assisted Photoacidity. In Hydrogen-Transfer Reactions, pp. 377-415. https://doi.org/10.1002/9783527611546.ch12.

S4. Heller, S.T., and Silverstein, T.P. (2020). pKa values in the undergraduate curriculum: introducing pKa values measured in DMSO to illustrate solvent effects. ChemTexts *6*. 10.1007/s40828-020-00112-z.

S5. Berton, C., Busiello, D.M., Zamuner, S., Solari, E., Scopelliti, R., Fadaei-Tirani, F., Severin, K., and Pezzato, C. (2020). Thermodynamics and kinetics of protonated merocyanine photoacids in water. Chem. Sci. *11*, 8457-8468. 10.1039/d0sc03152f.
